# Population Structure and Domestication Revealed by High-Depth Resequencing of Korean Cultivated and Wild Soybean Genomes^†^

**DOI:** 10.1093/dnares/dst047

**Published:** 2013-11-21

**Authors:** Won-Hyong Chung, Namhee Jeong, Jiwoong Kim, Woo Kyu Lee, Yun-Gyeong Lee, Sang-Heon Lee, Woongchang Yoon, Jin-Hyun Kim, Ik-Young Choi, Hong-Kyu Choi, Jung-Kyung Moon, Namshin Kim, Soon-Chun Jeong

**Affiliations:** 1 Korean Bioinformation Center, Korea Research Institute of Bioscience and Biotechnology, Daejeon 305-806, Republic of Korea; 2 Bio-Evaluation Center, Korea Research Institute of Bioscience and Biotechnology, Cheongwon, Chungbuk 363-883, Republic of Korea; 3 Department of Bioinformatics, University of Science and Technology, Daejeon 305-806, Republic of Korea; 4 Department of Genetic Engineering, Dong-A University, Busan 604-714, Republic of Korea; 5 National Instrumentation Center for Environmental Management, Seoul National University, Seoul 151-921, Republic of Korea; 6 National Institute of Crop Science, Rural Development Administration, Suwon 441-857, Republic of Korea

**Keywords:** domestication, resequencing, soybean, variation

## Abstract

Despite the importance of soybean as a major crop, genome-wide variation and evolution of cultivated soybeans are largely unknown. Here, we catalogued genome variation in an annual soybean population by high-depth resequencing of 10 cultivated and 6 wild accessions and obtained 3.87 million high-quality single-nucleotide polymorphisms (SNPs) after excluding the sites with missing data in any accession. Nuclear genome phylogeny supported a single origin for the cultivated soybeans. We identified 10-fold longer linkage disequilibrium (LD) in the wild soybean relative to wild maize and rice. Despite the small population size, the long LD and large SNP data allowed us to identify 206 candidate domestication regions with significantly lower diversity in the cultivated, but not in the wild, soybeans. Some of the genes in these candidate regions were associated with soybean homologues of canonical domestication genes. However, several examples, which are likely specific to soybean or eudicot crop plants, were also observed. Consequently, the variation data identified in this study should be valuable for breeding and for identifying agronomically important genes in soybeans. However, the long LD of wild soybeans may hinder pinpointing causal gene(s) in the candidate regions.

## 1. Introduction

Soybean is an important worldwide plant source of dietary protein and oil, and its capability of nitrogen fixation during symbiosis with rhizobia plays an important role in establishing sustainable agriculture systems. Cultivated soybean (*Glycine max*) is thought to have been domesticated from wild soybean (*Glycine soja*) with distribution in East Asia, including Korea, Japan, and most parts of China, as early as 7000–9000 years ago,^1^ although the exact domestication location(s) are under debate.^1,2^ Soybean has undergone radical phenotypic changes in the seed size, colour, shattering, seed dormancy, flowering time, and plant architecture during the process of domestication. Localization of the major causative genes, responsible for these traits, would facilitate improvements in soybean using marker-assisted breeding. Genome-wide association studies (GWAS) of the diverse soybean germplasm may offer the potential to rapidly resolve complex traits to gene-level resolution, as in maize and *Arabidopsis*. However, GWAS take years to construct segregating populations and require a high density of genome-wide markers. Association mapping is also prone to miss causative gene alleles, thereby requiring additional sequence-level mapping.^3^ The availability of a comprehensive genome variation catalogue for both cultivated and wild soybeans would greatly facilitate the identification of functional variations in elite varieties by comparing the genomic variations in an elite variety with data from the controls. Resequencing analyses of major crop plants, including soybean, rice, and maize, using a low-depth sequencing coverage have been reported recently.^4–7^ However, the strategy of pooling many accessions for variation calling might miss rare variants, as indicated in a dense resequencing study of 50 diverse rice accessions^8^ and deep *Arabidopsis* sequencing studies.^9,10^ Here, we provide dense variation data based on an analysis of high-depth resequencing data of a diverse group of 10 cultivated and 6 wild soybean genomes sequenced to >14× mean depth. Thus, our results will likely be useful for marker-assisted breeding and sequence-level gene mapping of soybean.

## 2. Materials and methods

### 2.1. Sampling

All samples were grown in greenhouses and in the field for morphological confirmation.

### 2.2. Sample preparation and sequencing

Seeds of each of soybean accessions (Supplementary Tables S1 and S2) were germinated in a dark chamber at 25°C in a pot. After primary leaves of the germinated seedlings opened, all etiolated shoots of the seedlings except their cotyledons were collected to extract genomic DNA. Sequencing libraries were constructed according to the manufacturer's instructions (Illumina, San Diego, CA, USA). By applying the Illumina HiSeq2000 platform, we generated 305 Gb of 101 bp paired-end short reads from 15 samples with insert sizes of 237–336 bp (Supplementary Table S3). The short-read sequence data are deposited into the European Nucleotide Archive under accession number ERP002622. We imported 39.8 Gb of the 76 bp paired-end reads for IT182932, which was reported previously by Kim *et al.*^11^ Over 94% of the reads (86.3% for IT182932) were mapped onto the whole Williams 82 reference genome. A total of 86.3–90% of reads were mapped onto the chromosomes (Gm01–Gm20) except reads of IT182932 (76.1%). After removing duplicated reads, we used 78.9–88.5% (71.9% for IT182932) of the sequenced reads for downstream analysis (Supplementary Table S3).

### 2.3. Genome size estimation

The estimation of genome size for each accession was computed based on counting of occurrences of *K*-mers (substring of length *K*). We estimated the genome size using JELLYFISH 1.1.5^12^ with *K*-mer as 17 bp JELLYFISH counted 17-mer frequencies per depth. As the peak of 17-mer frequency (*M*) in reads is correlated with the real sequencing depth (*N*), read length (*L*), and *K*-mer length (*K*), their relationship can be expressed in a formula used in the Panda genome project: *M* = *N* × (*L* – *K* + 1)/*L*.^13^ Then, the estimated genome size was obtained by dividing the total sequence length by the real sequencing depth.

### 2.4. Reads mapping

Paired-end reads were mapped onto the Williams 82 reference genome (Glyma1) using Burrows Wheeler Aligner (version 0.5.9) with default options.^14^ The reference genome sequence was downloaded from the JGI genome portal (ftp.jgi-psf.org/pub/compgen/phytozome/V9.0/Gmax/assembly/Gmax_189.fa.gz, accessed on 10 November 2013). *Glycine max* chloroplast genome (NC_007942) was separately downloaded from the NCBI ftp site (ftp://ftp.ncbi.nlm.nih.gov/genomes/Glycine_max/CHR_Pltd/gma_ref_V1.0_chrPltd.fa.gz, accessed on 10 November 2013) and included in the reference genome. After initial mapping of the raw reads onto the reference genome, we screened the reads that were mapped as unplaced scaffolds and the unmapped reads. Aligned reads considered to be PCR duplicates were removed using the MarkDuplicates in the Picard software package 1.48 (http://picard.sourceforge.net/, accessed on 10 November 2013). Mate information was re-synchronized using the Picard FixMateInformation tool. Alignments around the small indels were re-aligned with IndelRealigner, and base-pair quality scores (QUAL) were recalibrated with CountCovariates and TableRecalibration in the Genome Analysis Toolkit (GATK; version 1.0.5974).^15^ The realigned, recalibrated SAM (Sequence Alignment/Map) files produced by these processing steps were used for single-nucleotide polymorphism (SNP)/indel detection and for all alignment-related statistics, such as allele counts.

### 2.5. SNP and indel detection

Individual genotyping was performed to identify SNPs and indels for each of the 16 samples. Then, multi-sample SNP genotyping was performed to compare the genotypes on the variant positions, where all samples were able to be genotyped. UnifiedGenotyper in GATK^16^ was used to generate the initial SNP and small indel calls. The SNP/indels were called with standard call confidence (-stand_call_conf) set to 30.0 and standard emit confidence (-stand_emit_conf) set to 10.0. An indel model (-glm INDEL) was enabled in indel calling. To identify high-quality variants, the initially called SNP/indels were evaluated with a Gaussian mixture model that was built with known soybean variants (dbSNP; version 133), and outliers were discarded. To reduce the SNP/indel false discovery rate, raw variant calls were filtered using VariantFiltration in GATK for the following annotations: QUAL of ≤50.0, depth of coverage (DP) ≤5, call quality divided by depth (QD) ≤5, mapping quality (MQ) ≤30, strand bias (SB) greater than or equal to −1.0, MQ zero reads (MQ0) ≥4, and MQ0 divided by depth (MQ0/DP) ≥0.1. We designed random primer pairs across the genome and performed Sanger sequencing to validate genotypes called using our Illumina data. Agreement between locations of SNPs/indels mapped in the Hwangkeum × IT182932 population,^17^ and SNPs/indels called in this study was also examined for validation.

### 2.6. Identification of non-reference genes

We assembled the unmapped reads from each sample into contigs using SOAPdenovo.^18^ Default parameters were used and only contigs, not scaffolds, were constructed. We first assembled the unmapped reads separately in each accession, and contigs shorter than 2 kb were excluded to identify novel sequences. We then used the self-alignment approach to exclude the redundant sequences. In total, we identified 10 035 contigs with a total length of 31.5 Mb (Supplementary Table S6). In a previous analysis of IT182832 short reads using different methods, Kim *et al*.^11^ reported a total length of 8.3 Mb much longer than 0.83 Mb obtained in this study. However, a total length of contigs longer than 300 bp from IT182932 in the current analysis was 9.1 Mb, indicating that this difference likely reflects different methods and cut-off criteria. We conducted *de novo* gene annotation with AUGUSTUS^19^ for the 10 035 contigs. After annotation, we excluded the redundant genes that were assembled in different accessions. Only one copy of the genes with >90% identity and >90% coverage by BLAT was retained. In total, we annotated 1363 possible genes *de novo* containing ≥100 amino acids. Then, we used BLASTP^20^ to compare the candidate novel genes against the NCBI non-redundant (nr) database (>60% identity and 60% coverage). Then, we further performed functional annotation using InterProScan^21^ and HMMer3.^22^

We attempted to validate some of these gain genes using a PCR amplification method. We chose 51 gain genes (transposon genes excluded) from the cultivars Williams 82K (*n* = 4) and Hwangkeum (8), as well as the wild soybean IT162825 (39). PCRs were performed using the cultivated soybeans Williams 82K and Hwangkeum, as well as the wild soybeans IT182932 and IT162825 as templates. In addition to singlet PCR using a pair of primers designed from each of the chosen genes, we performed multiplex PCR by adding a pair of primers designed from the soybean actin gene to be certain of the absence of the expected PCR products. The actin gene primers were: forward 5′-TGGACTCTGGTGAT GGTGTC-3′ and reverse 5′-CTCCAATCCAAACACTGTA-3′.^23^

### 2.7. Identification of gene loss events

To identify gene loss events, we first discovered medium-size deletion (ranging from 200 to 300 000 bp) sites in the Williams 82 reference genome using Genome STRiP 1.0.3.^24^ Genome STRiP identifies structural variations in populations by using information from read pairs with unexpected alignments as well as analysis of read depth. We ran the Genome STRiP with default parameters for 15 accessions except IT182932. Calls ‘not detected’ in more than three accessions were excluded. This reduced false positives due to low depth of resequencing data at some regions or due to errors in the reference genome sequence. As a result, we identified 10 928 medium-size deletions in various accessions and then examined if these deletions were associated with gene loss events based on gene annotations from the soybean reference v1.1 gene set, which was downloaded from the SoyBase website (http://www.soybase.org, accessed on 10 November 2013). Validation of the gene loss events was performed by designing pairs of primers that amplify 200–300 bp DNA fragments flanking the deletion sites. We randomly chose 55 deletions of various sizes and used PCR to experimentally validate the deletions.

### 2.8. Gene ontology term enrichment analysis

Gene ontology (GO) term enrichment analysis was performed on the non-reference (or gained) gene set, the gene set associated with gene loss events, and the identified candidate domestication or improvement gene set. We first loaded protein sequences of the gene sets into Pathway Studio ver. 9 (Elsevier). The enrichment analyses were performed using the ResNet Plant database (version 4.0) provided by this software. The identified orthologues using the BLAST best reciprocal hit method were used for the enrichment analysis. The algorithm returned a probability value (*P*) for the statistical significance test of gene enrichment between the input gene set and GO group. We considered a GO term to be enriched if the *P*-value was <0.001.

### 2.9. Construction of phylogeny

SNPs were used to calculate the genetic distances between different accessions. The neighbour-joining method^25^ was applied to construct the phylogenetic tree based on the *p*-distance method and the bootstrap confidence analysis with 1000 replicates. The trees were drawn to scale, with branch lengths in the same units as those of the evolutionary distances, which are in the units of the number of base differences per site. The constructed phylogenetic tree was plotted using MEGA5.^26^

### 2.10. Population structure inference

We used the STRUCTURE version 2.3.2,^27^ which is based on the maximum-likelihood method, to investigate the population structure across different values of *K* (number of putative ancestral clusters of allelic similarity). We used an admixture model with correlated allele frequency^28^ to assign individuals into *K* clusters. A 10,000 burn-in period of Chain Monte Carlo searches followed by 20,000 replicate runs were performed at each *K* from 2 to 7. We conducted STRUCTURE analysis for 17 accessions consisting of the reference genome (Williams 82), 10 cultivated, and 6 wild soybeans as well as an expanded set consisting of these 17 and 31 accessions from Lam *et al.*^5^

### 2.11. Calculation of linkage disequilibrium

The correlation coefficient (*r*^2^) of alleles was calculated to measure linkage disequilibrium (LD) levels in both the wild and cultivated soybeans using the Haploview software.^29^ The parameters were set as follows: maximum intermarker distance for LD comparisons (maxdistance) to 1000, output to pairwise LD text table format (dprime), minimum minor allele frequency (minMAF) to 0.1, and minimum Hardy-Weinberg *P*-value (hwcutoff) to 0.001. The average *r*^2^ values were calculated for pairwise marker distances, and the values were smoothed using the LOWESS function of R for both cultivated and wild soybean populations.

### 2.12. Estimation of population parameters and detection of putative artificially selected genes

Population statistics *π*, *θ*_w_, and *F*_ST_ were calculated using in-house custom scripts, as described previously.^30–32^ The analysis using the reduction of diversity (ROD = 1 − *π*_cul_/*π*_wild_) introduced by Xu *et al*.^8^ was adapted to our samples. ROD values were calculated based on the ratio of diversity in the cultivated soybeans to the diversity in the wild soybeans (*π*_cul_/*π*_wild_) in 20% sliding windows along the entire genome. We performed this analysis using windows of 10, 50, 100, 150, 200, and 500 kb. The windows with >0.98 ROD values were picked out as candidate selective sweep regions, and genes in these regions were identified as putative genes under selection. Windows in multiple pericentromeric regions in our initial analysis showed high ROD values, as observed in a maize resequencing study.^7^ Because pericentromeric regions harbour few genes and correlations between genetic map and reference genome sequence in these regions are generally low,^17^ we masked pericentromeric regions from further analysis (Supplementary Table S4). In addition, we masked windows in the bottom 1% of SNP frequency.

## 3. Results and discussion

### 3.1. Sequencing and mapping

**Figure 1.**
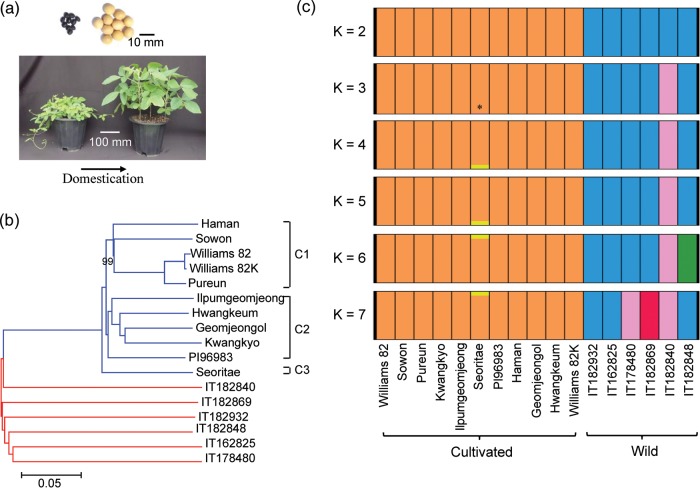
Soybean population structure. (a) Changing morphology of domesticated soybean (left) and its wild relative (right). (b) Neighbour-joining phylogenetic tree of soybean nuclear genomes based on the high-quality SNPs, with the evolutionary distances measured by the *p*-distance. All branches except one denoted were supported by 100% bootstrap values from 1000 bootstrap replications. Taxa in the neighbour-joining tree (right) are represented by different colours: wild (red) and cultivated (blue) soybeans. Cultivated soybeans were tentatively grouped into C1, C2, and C3. (c) Bayesian clustering of samples using the STRUCTURE program. Each accession is represented by a vertical bar and each colour represents one population. An asterisk indicates a narrow pink segment, which is visible when enlarged. The mean value of ln-likelihood when *K* changed from 2 to 7 was −41525152, −37378506, −37280892, −32931343, −34405078, and −37518839, respectively.

Wild and cultivated (domesticated) soybeans are classified into distinct species: cultivated soybean is *G. max* and its wild relatives are *G. soja*.^33^ However, a cultivated plant and its wild relative can normally cross and produce a fertile F_1_ hybrid generation despite distinctive phenotypes (Fig. 1a). Furthermore, *G. soja* is the only wild member of the subgenus *Soja* distributed in East Asia.^34^ Thus, this crop and its wild ancestor have been proposed to be more appropriately classified as *G. max* subsp. *max* and *G. max* subsp. *soja*, respectively,^35^ although most of the current literature and this study still use *G. soja* as the scientific name of the annual wild soybean. With this consideration, we use the term ‘cultivated’ and ‘wild’ soybeans to refer to *G. max* and *G. soja*, respectively, throughout this study to avoid the ambiguity of the current nomenclature.

We selected 10 cultivated soybean accessions that represent green, sprout, sauce-tofu, and cooking-with-rice soybeans as four groups classified in terms of food use in Korea (Supplementary Table S1). Among these cultivars, four are considered to be landraces collected in Korea (Supplementary Fig. S1) and six are improved varieties. To strictly control the quality of our sequencing and SNP calling, we also included Williams 82K,^17^ a variant of the Williams 82 strain, which was used to generate the reference soybean genome sequence.^36^ All improved soybeans, except Williams 82 which was bred by backcross breeding in the USA, were bred using a pedigree breeding method in Korea (Supplementary Table S2). Parental lines of improved varieties originated from China, Japan, Korea, Taiwan, and the USA. Although some of the parental lines share common ancestor(s) in their pedigrees, a unique parent in each of the five improved varieties should contribute ∼50% genetic variation from a unique landrace through this breeding method. Six wild accession samples were collected according to the geographic distribution of the wild soybean in Korea (Supplementary Table S1 and Fig. S1). As Korea is located in the centre of the ancient soybean cultivation and wild soybean distribution areas and has a long history of soybean use as food, we hypothesized that intensive sequencing of representative Korean soybean genomes might reveal the genetic diversity of soybean.^37^

We sequenced these 15 chosen accessions to >17× coverage (raw data) using an Illumina HiSeq2000 instrument except the wild soybean IT182932 (Supplementary Table S1). Genome resequencing analysis of IT182932 was reported by Kim *et al.*^11^ Raw reads of 39.8 Gb of the IT182932 genome produced by Illumina-GA were re-analysed in this study. Variation calling results of Williams 82K, Hwangkeum, and IT182932 were partly reported in our previous study, which compared soybean genetic and sequence-based physical maps.^17^ However, population-level analysis using dense variation data of these 10 cultivated and 6 wild accessions is first reported in this study. After removing duplicate reads (Supplementary Table S3), the final mean depth was >14× for all accessions and >97% of the reference genome was covered by more than one read and >94% by more than five reads for all accessions, but IT182932 (96.1 and 85.4%, respectively) (Supplementary Table S1). Homozygous variation callings required a minimum of five reads. The genome coverages were higher and much less variable than those of a similar study for rice accessions that ranged from 79 to 94%,^8^ indicating that, relative to rice, soybeans contain smaller portions of individual-specific or diverged sequences. The high variability in mapping rates among rice accessions is likely due to significant genome size differences among rice subspecies.^38^ The similarity of genome sizes between cultivated and wild soybean accessions was further tested by estimating the genome sizes of soybean accessions using a distribution of 17-mer frequency in the usable sequencing reads to determine the sequencing depth.^13^ We obtained estimated genome sizes (±SEM) of 1107 ± 11 and 1106 ± 22 Mb from 10 cultivated and 6 wild soybeans, respectively (Supplementary Table S5). The two estimated values were not significantly different (*t*-test, *P*-value = 0.990; *F*-test, *P*-value = 0.211). Thus, relative to *Oryza sativa* (rice) that is divided into several subspecies, the cultivated and wild soybeans that are widely divided into two different species, *G. max* and *G. soja*, should be regarded as a single species. The mean value of 1106 Mb from 16 soybeans was also similar to that of 1115 Mb estimated by flow cytometry,^39^ suggesting that the Williams 82 sequence assembly of 955 Mb^36^ may miss ∼150 Mb.

### 3.2. Identification of gene gain and loss events across the soybean genome

High-depth resequencing data allowed us to identify gene gain and loss events across the soybean genome. We assembled unmapped reads for each accession into contigs (Supplementary Table S6) and then used *de novo* gene prediction to annotate 1326 putative genes in the contigs, of which 345 had homologues in the NCBI nr database and 343 of these had homologues in plants (Supplementary Table S7, S8, and Supplementary Data Set 1). Interestingly, of the eight genes that we annotated in two sequences identified as the missing sequences of the Williams 82 genome assembly in our previous report,^17^ seven had >85% similarity in these 343 genes and four were identical in these 343 genes, indicating that many of the unmapped genes represent genes from missing sequences in the Williams 82 genome assembly. To test whether some of these unmapped genes are found only in a certain accession, we chose 51 such ‘novel’ genes for PCR validation. When two cultivated and two wild accessions were tested, the proper PCR product size for all 51 was amplified in one or more of the four tested accessions (Supplementary Fig. S2). Of the 51, 20, including four from Williams 82K, were amplified in all four accessions and 31 were absent in one or more of the tested four, indicating that although some of our *de novo* assembled genes reflected incompleteness of the current Williams 82 genome assembly, >60% were gain genes whose presence/absence in the soybean population were variable (Supplementary Table S8). Interestingly, the presence/absence patterns of 12 non-reference genome genes agreed to those predicted from our *de novo* assembly. The 343 gain genes consist of 133 characterized gene homologues, 108 uncharacterized gene homologues, and 102 transposon-related genes, indicating that some of these novel genes may have arisen from the movement of transposable elements, which commonly create incomplete pseudogenes in plants. However, in the GO analysis using the Pathway Studio (Elsevier), we found no GO term enriched by ≥2 genes in the 133 genes.

In addition to the novel non-reference genome genes, we also identified genes absent in some accessions relative to the reference genome. We used the information of discordant paired-end reads at the deletion sites to collect evidence supporting these gene loss events. We identified 10 928 medium-size deletions between 229 and 308 146 bp (Supplementary Table S9 and Supplementary Data Set 2). Validation of the deletions by PCR suggested a false-positive rate of 26.6% in the dataset, which is higher than that (1 of 9) obtained with analysis of rice genome^8^ (Supplementary Fig. S3 and Table S9). Our close examination of the false-positive deletions showed that many of them correspond to the sites containing ambiguous 100–1000 N bps in the reference assembly as observed in our previous study.^17^ However, because some of the N-stretch-containing sites were shown to be positives in our validation, the predicted N-stretch-containing deletions were retained in our current data set (Supplementary Data Set 2). Of the deletions, 1737 were associated with gene loss events. The GO analysis of the 1737 lost genes yielded many enriched GO terms. Of the 30 significantly enriched GO terms in the biological process category, 12 were related to seed development, germination, and flower/leaf development reminiscent of the domestication-related traits (Supplementary Table S10). Domestication loci tend to show altered allele frequency.^40^ Most of the gene loss events in the 12 enriched GO terms did not show differential frequency between the cultivated and wild soybeans. However, 10 events showed distinct occurrence in the cultivated or wild soybeans (Supplementary Table S11), thereby suggesting that they may be candidate domestication genes. Functional studies of the 10 lost genes have been mostly performed in *Arabidopsis*, which is not a crop species, and only one gene, *PAP15* (*Glyma11g36510*), has functionally been studied in several crop species including soybean.^41,42^ Overexpression of *Arabidopsis PAP15* in soybean increased phytase activity in the soybean tissue.^41^ As numerous studies, including a recent report from Maupin and Rainey,^43^ have shown a significant relationship between phytate content and germination percentage of soybean seeds, which is a domestication-related trait,^44^ our result indicated that *PAP15* is a strong domestication candidate gene.

### 3.3. Variation across the soybean genome

We identified ∼9 million candidate SNPs in all 16 accessions by mapping reads for each accession to the reference genome sequence (NCBI dbSNP [ss7843006444–ss792422648]; viewable via our Soya Genome Browser (http://soya.rna.kr, accessed on 10 November 2013)). To obtain high-quality SNPs for population analyses, we excluded SNPs with missing data in any of the 16 accessions, as these would make subsequent inferences unreliable, yielding a final total of 3 871 469 high-quality SNPs (Table 1 and Supplementary Table S12). This represents the largest high-quality SNP dataset obtained in soybeans. In a low-depth resequencing analysis of 31 soybean accessions, Lam *et al.*^5^ reported 966 612 high-quality SNPs. Although most of the SNPs were located in intergenic regions, the number of genic SNPs, 788 809 (20.4%), are almost comparable with the total number of SNPs reported by Lam *et al.*^5^ A total of 1 109 412 non-singleton SNPs, which occurred in more than two accessions as homozygous SNPs, were identified (Supplementary Data Set 3). The non-singleton (common) SNPs can serve as a resource to identify tag SNPs that will be needed to capture most haplotype variation for GWAS and designing useful breeding programmes in soybean.^45^ Thus, our data may be useful to identify important soybean genes by serving as molecular markers for designing soybean SNP arrays and for breeding. Among the 3.8 million high-quality SNPs, 1 687 232 were found in cultivars. A large proportion (1 106 593; 65.6%) of these SNPs was also found in wild soybean accessions, indicating that most genetic variation in cultivated soybean is derived from variation in wild soybean. Of the remaining 580 639 (34.4%) cultivar-specific SNPs, some might have been false positives due to the relatively small wild soybean sample size. We identified 3 290 830 high-quality SNPs in the six wild soybeans, of which 66.4% were wild-specific. Of the SNPs in the wild soybeans, 49.9% were a single wild accession-specific, while 36.2% of the SNPs in the cultivated soybean were a single cultivated accession-specific (Supplementary Fig. S4). Collectively, our results suggested that wild soybean has a much more diverse gene pool than cultivars and thus, may contain useful genetic resources for improving soybean.

**Table 1.** Summary of SNPs and indels variations for cultivated and wild soybeans obtained from individual or multi-sample genotyping

Group	Sample size	SNP			Indel		
		Total	Genic	CDS^a^	Total	Genic	CDS
Individual genotyping							
Cultivated	10	4 182 059	618 493	139 107	799 470	131 032	7269
Wild type	6	7 626 486	2 276 394	252 245	1 447 750	246 653	13 003
Total	16	9 028 250	1 138 197	296 648	1 769 260	294 390	15 764
Multi-sample genotyping							
Cultivated	10	1 687 232	352 771	78 172	225 609	57 543	3972
Wild type	6	3 290 830	675 710	147 121	430 564	108 560	6824
Total	16	3 871 469	788 809	173 293	499 865	125 289	8222

^a^Variations in coding sequences (CDS) of representative genes. As alternative transcripts were not included, the numbers presented should be regarded as approximate.

In addition to SNPs, we also detected 1 769 260 candidate insertions (1–35 bp) and deletions (1–56 bp) (indels) by mapping reads with gaps allowed (Table 1) (NCBI dbSNP [ss7843006444–ss792422648]; Soya Genome Browser (http://soya.rna.kr, accessed on 10 November 2013)). After excluding indels with missing data in any of the 16 accessions, 499 865 high-quality indels were retained. We observed slightly more deletions than insertions. Single-base-pair indels were the most frequent, and numbers of longer indels decreased abruptly (Supplementary Fig. S5). Similar to trends observed for SNPs, the wild soybeans contained more indels than the cultivated soybeans (Supplementary Fig. S4 and Table 1), and rare variants comprised a large proportion of the total indels with ∼73.2% of indels found in less than four accessions (Supplementary Fig. S4). Among the ∼0.5 million high-quality indels, we identified 86.1% (430 564) of them in the wild soybeans, of which 79.8% were wild-specific. Most of the indels were located in intergenic regions, and 1.6% (8222) of the indels were located in coding sequences.

To validate the variation calling results, we designed primer pairs from genomic regions of nine randomly selected genes as well as of two candidate domestication genes described below and performed Sanger sequencing. We sequenced 24.5 kb of soybean genomic DNA from each of the 16 accessions (GenBank: KF220802–KF221057). Of the 112 high-quality SNPs and 31 high-quality indels, except a Glyma20g32540 genomic region that showed unusually the high number of heterozygous variations, 109 (97% accuracy) and 28 (90% accuracy), respectively, were validated. Locations of SNPs/indels mapped in the Hwangkeum × IT182932 population^17^ and SNPs/indels called in this study agreed to each other. Our close examination of the *Glyma20g32540* genomic sequence in the Williams 82 reference genome assembly suggested that the high heterozygous variation callings were caused by that reads from its homeologous genomic region containing *Glyma10g35017*, part of which was ambiguous with one 100-N bp, were mapped onto the Glyma20g32540 region. This notion was further confirmed by determining the ambiguous sequence by sequencing PCR products encompassing the 100-N-bp region. These results indicated that, due to the missing or ambiguous parts of the reference genome sequence, some degree of heterozygous SNP callings in these regions are inevitable. These validations also served to confirm the high quality of our SNP and indel dataset.

### 3.4. Population structure of cultivated and wild soybeans

To examine the genetic population structure and relationships among the cultivated and wild soybeans, we constructed a neighbour-joining tree^25,26^ based on the 3 329 657 high-quality SNPs after excluding SNPs with heterozygous genotypes in some of the 16 accessions as well as SNPs identified in the unanchored 17 Mb scaffolds. The cultivated and wild soybeans formed two clearly separated subclades (Fig. 1b). Seoritae was most closely related to the wild soybeans in our phylogenetic tree. Pairwise distances between each pair of the wild soybeans were greater than those between any pairs of the cultivated soybeans, indicating that geographic distances of the collection sites in Korea are large enough to prevent intermixing of those wild soybeans analysed in this study, and that diversity between any pair of the collected wild soybeans is greater than those among all cultivated soybeans. When we compared pairwise distances between IT182848 and any of the wild and cultivated soybeans (Supplementary Table S13), the mean distance value between the IT182848 and wild soybeans is not significantly different from that between the IT182848 and cultivated soybeans (*t*-test, *P* = 0.324). Taken together, our phylogenetic tree analysis suggested that the cultivated soybean population is a subclade of the wild soybean population rather than an independent species or subspecies.

To further investigate the population structure, we used the STRUCTURE program,^27^ which estimates individual ancestry and admixture proportions assuming that *K* populations exist based on a maximum-likelihood method.^46^ We analysed the data by increasing *K* (the number of populations) from 2 to 7 (Fig. 1c). We found a division between the cultivated and wild soybeans for *K* = 2. For *K*'s ranging from 3 to 7, the variety of Seoritae consistently appeared to be slightly different from the remaining cultivated soybeans. Although numbers of the groups for *K* > 5 are smaller than that of *K* likely because of small population size, the number of groups within the wild soybean group increased with an increasing number of *K* up to three groups at *K* = 7. These results were consistent with the data from our phylogenetic analysis in that the cultivated soybeans are largely divided into three subgroups within a subclade (which was supported by results with an additional 31 soybeans described below), and each of the wild accessions contains more accession-specific SNPs than any of the cultivated accessions.

The evolutionary history of soybean and the range of diversity of our resequenced cultivated soybeans were further revolved by incorporating the SNP data (downloaded from the BGI soybean resequencing ftp site; ftp://public.genomics.org.cn/BGI/soybean_resequencing/, accessed on 10 November 2013) from 17 wild and 14 cultivated soybean genomes resequenced at a low depth (∼5×) reported by Lam *et al.*^5^ into our current SNP data set. We then extracted SNPs covered by all 31 accessions without the missing data or heterozygous genotypes. By intersecting these SNPs with the set of 3.3 million high-quality SNPs identified in our 16 accessions, we obtained 208 684 SNPs that could be used to analyse all 23 wild and 24 cultivated soybean accessions. The results again showed that cultivated soybeans represent a subclade of a large soybean clade, indicating that wild soybean is the direct progenitor of cultivated soybean (Supplementary Fig. S6). Excluding the four admixture accessions,^5^ which were also supported by our population structure analysis, the cultivated soybeans formed three well-defined subgroups, each of which contained at least one accession of the 10 accessions examined in this study. This indicated that the diversity level of the cultivated soybeans obtained in this study likely serves as a leverage for elucidation of the comprehensive diversity of the global cultivated soybeans. However, the six wild strains resequenced in this study were clustered into a single small clade, indicating that the diversity level of wild soybean in the East Asia is much greater than that obtained in this study.

### 3.5. Identification of genome regions most affected by selection during soybean evolution

Phenotypic traits associated with domestication syndrome that have undergone repeated artificial selection usually have a reduction in nucleotide diversity and altered allele frequency in the domestication loci,^40^ as parameters that have successfully been used to identify putative artificially selected genes in crops and domesticated animals including maize (e.g.^7,47^), rice,^8^ cattle,^48^ and dogs.^49^ Our large SNP dataset (>3.8 M) from both the wild and cultivated soybeans provides an opportunity to identify the selected genes by comparing the polymorphism levels between cultivated and wild accessions.

**Figure 2.**
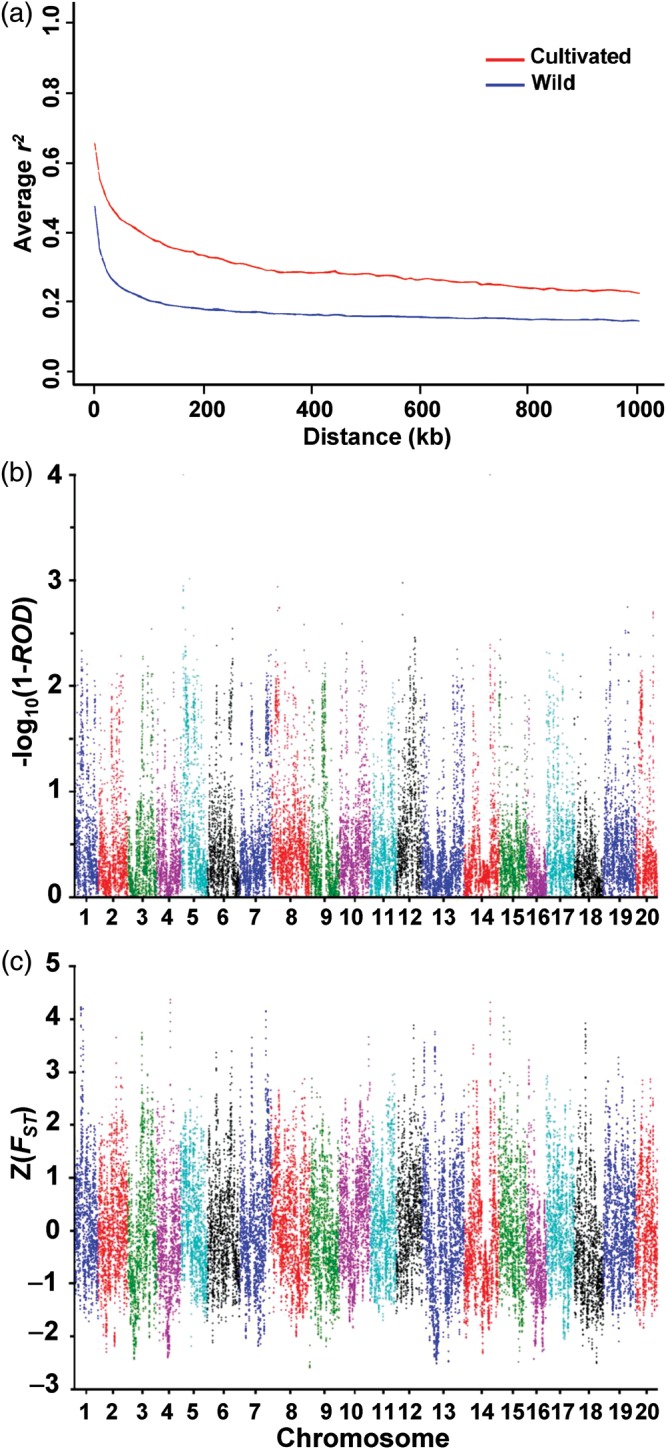
Genome-wide analysis of nucleotide diversity and selection. (a) LOWESS curves of LD decay patterns determined by squared correlations of allele frequencies (*r*^2^) against distance between polymorphic sites in cultivated (red) and wild (blue) soybeans. (b and c) Distributions of ROD values (b) and *Z*-transformed fixation index (*F*_ST_) values (c) for cultivated relatives to wild soybeans in 100-kb windows across the genome. ROD = 0.98 corresponds with *–*log_10_(1 − ROD) = 1.70. The chromosome number is indicated along the *x-*axis.

The processes of domestication, which is repeated artificial selection, can increase LD through the genome or in genomic segments flanking domestication loci.^50^ LD decayed to half of its maximum value at ∼120 kb for wild soybeans and at ∼340 kb for cultivated soybeans (Fig. 2a). These LD distances are quite similar to those (wild, ∼75 kb and cultivated, ∼150 kb) estimated by low-depth resequencing data of 17 wild and 14 cultivated soybeans.^5^ The LD distance of cultivated soybean is comparable with those of subpopulations of rice subspecies *japonica*, which ranged from 180 to 300 kb.^8^ However, LD distance of the wild soybeans is extremely longer than those of the wild plants analysed at the genome level to date, which are usually within <10 kb (e.g.^8,51,52^). This long distance is likely due to the selfing nature of wild soybeans unlike wild maize and rice species.^45^ Thus, our results suggest that although two to three times longer LD distance for the cultivated than that for wild soybean accessions indicated the existence of some degree of domestication or improvement bottleneck in the cultivated soybeans, the relatively long LD of the wild soybeans may allow us to easily identify approximate domestication or improvement regions using whole-genome resequencing data. However, it would be more difficult to pinpoint causal domestication gene(s) in the identified regions in soybean relative to rice or maize.

We sought to identify regions with significantly lower levels of polymorphisms in the cultivated soybeans relative to the wild soybeans to detect selective sweeps driven by artificial selection.^53^ Thus, we used an ROD (ROD = 1 − *π*_cul_/*π*_wild_) method^8^ to identify regions of the genome most affected by selection during soybean evolution. ROD values were calculated based on the ratio of diversity in the cultivated soybeans to diversity in the wild soybeans (*π*_cul_/*π*_wild_) in 20% sliding windows along the entire genome except pericentromeric regions (Supplementary Table S4). We performed this analysis using windows of 10, 50, 100, 150, 200, and 500 kb with >0.98 as a cut-off ROD value (Fig. 2b and Supplementary Fig. S7). The estimated genome diversity levels using *π* and *θ*_w_ parameters^31^ were >2-fold lower in the cultivated soybeans (*π* = 0.46 × 10^−3^ and *θ*_w_ = 0.46 × 10^−3^) compared with those in the wild soybeans (*π* = 1.08 × 10^−3^ and *θ*_w_ = 1.15 × 10^−3^). The windows having >0.98 ROD values that showed extremely low diversity in cultivars might have experienced cultivar-specific selective sweeps. Adjacent windows with >0.98 ROD values were grouped into candidate domestication regions (CDRs), which likely represent the effect of a single selective sweep, as previously suggested.^7^ The detected CDRs in each of the 10, 50, 100, 150, 200, and 500-kb windows covered ∼15.5, 9.8, 7.2, 5.7, 4.4, and 1.6%, respectively, of the analysed soybean genome. Thus, the 100-kb windows showed genome coverage similar to that (∼7.6%) of CDRs obtained in a recent maize study.^7^ Portions of the detected chromosomal regions with significant ROD values in different windows overlapped with each other. The regions detected by larger windows were a subset of those detected by smaller windows except for slight differences in window margins (Supplementary Fig. S7). We further calculated the divergence index *F*_ST_ using the same sliding window approach. We found that >80 and >50% of the windows with >0.98 ROD values were found in 25 and 10% upper tails, respectively, of the *F*_ST_ distribution in the larger than 100-kb windows (Supplementary Table S14 and Fig. S8). Relative to the extreme 90% distribution of *japonica* ROD regions falling in the 5% right tail of the genome-wide *F*_ST_ distribution,^8^ broader *F*_ST_ distribution of the soybean ROD regions is likely due to much longer LD distances (120 kb) of the wild soybeans relative to those of the wild rice and maize (<10 kb), as shown in our LD estimation described above. Collectively, our results, including the LD estimation, genome coverage of CDRs, and comparison between distributions of *F*_ST_ and ROD values, suggested that, in this study, the 100-kb window would maximize the precision for detecting the candidate regions under selection.

We focused analyses on the 206 CDRs identified using the 100-kb windows, which contained 3068 genes (Supplementary Table S15). CDRs contained an average of 14.9 genes and had a mean size of 189 kb. The longest fragment extended to 880 kb, and the median was 140 kb. We considered all genes in these CDRs to be candidates of artificially selected genes. It is likely that many of these genes were not themselves subjected to selection, but rather hitchhiked along with the actual gene targeted for artificial selection. More analyses, including transgenic experiments, are needed to identify the actual selected genes.

**Figure 3.**
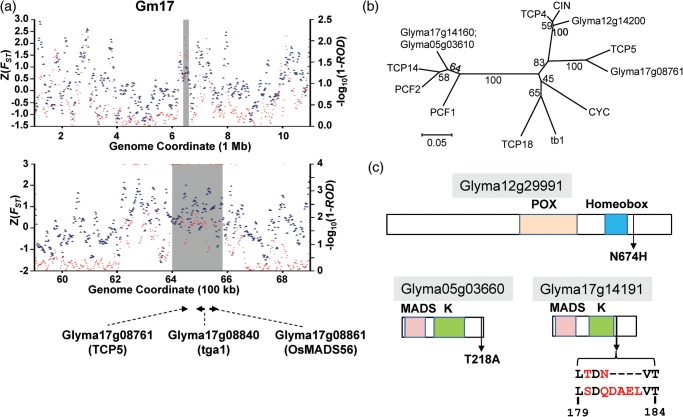
Features of candidate domestication genes homologous to cloned canonical domestication genes. (a) ROD (red), and average fixation index, *F*_ST_ (blue), plotted for 100-kb windows across a 10-Mb region (upper panel) or for 10-kb windows across a 1-Mb region (lower panel) of chromosome 17, which harbours a cluster of candidate domestication genes homologous to cloned canonical domestication genes. Strong candidate domestication genes in the region are shown below (a). Grey boxes indicate a 180-kb chromosomal region having >0.98 ROD values in 100-kb windows and its corresponding region in 10-kb windows. For simplicity, *–*log_10_(1 − ROD) values of ≥4 are shown as corresponding with *–*log_10_(1 − ROD) = 4. (b) Neighbour-joining phylogenetic tree showing relationship among soybean TCP family proteins, which appeared in CDRs, and functionally characterized representative members of other species, which were described by Martín-Trillo and Cubas.^60^ The percentage of bootstrap samples is shown to indicate the reliability for branching. Only the TCP domain was used for the analysis. See Supplementary Fig. S9 for the phylogenetic relationship among all predicted TCP proteins of soybean and representative members of other species. (c) Structure of three candidate domestication proteins showing conserved domains (coloured boxes) and positions of amino acid substitutions by nsSNPs fixed in wild soybeans. Glyma12g29991 is homologous to qSH1 and Glyma05g03660 and Glyma17g14191 are homologous to OsMADS56. POX is a functionally unknown domain named ‘associated with HOX’; homeobox is BEL1-type homeobox; MADS is SRF-type MADS box; *K* is *K*-box region.

No canonical soybean domestication gene has been cloned yet. The genes annotated in the detected CDRs should prove useful, both for dissecting known quantitative trait loci (QTL) and identifying novel candidate domestication genes. When we searched for homologues of the domestication genes characterized in other crop plants such as rice, maize, and tomato, homologues of several canonical domestication genes including *tga1*, *qSH1*, *fw2.2*, *PROG1*, *DAG1*, *DAG2*, *Tunicate1*, and *OsMADS56* were successfully identified in our putative artificial selection gene set (Supplementary Table S16). Although the majority of those canonical domestication genes have been functionally characterized in monocot crops distantly related to the soybean, most of CDRs containing their soybean homologues are, to our surprise, associated with QTL hotspots for domestication-related traits, such as plant height, leaflet shape, seed weight, pod dehiscence, and pod number. For example, *Glyma17g08840* homologous to *tga1* and *Glyma17g08861* homologous to *OsMADS56* are located in the same CDR on Gm17 and are associated with QTL that regulate multiple domestication-related traits, including leaflet length, plant weight, seed weight, and yield (Supplementary Table S16 and Fig. 3a). At the same CDR, we observed a putative transcription factor Glyma17g08761 containing TCP (TEOSINTE BRANCNED 1, cycloidea and PCF) domain characteristic of the maize gene *tb1* that regulates the plant and inflorescence structure, although its TCP domain showed less degree of amino acid identity (∼50%) than that of several other soybean TCP-containing proteins (∼70%) to that of the tb1 protein (Fig. 3b). Thus, consistent with the multiple QTL reports by several studies, the CDR on Gm17 appears to be a hotspot for the domestication genes. However, its duplicated chromosomal segment from palaeopolyploidization,^36^ which is corresponding to the north end of Gm05, was not detected as a CDR.

Many of causal mutations in the cloned domestication genes are amino acid changes, although regulatory changes almost equally contribute to causal mutations.^53^ To further characterize the genomic impact of domestication, we examined whether variations in coding sequences of the nine soybean genes homologous to canonical domestication genes described above changed the amino acid sequences of the expressed proteins (Table 2). Of the nine genes, three including Glyma16g02550.3, Glyma08g07260.3, and Glyma17g08861.1 did not contain any non-synonymous SNP (nsSNP). The remaining six genes contained various numbers of nsSNPs with different minor allele frequencies, most of which are <0.5. Interestingly, each of Glyma12g29991.3 (homologous to qSH1) and Glyma05g03660.8 (homologous to OsMADS56) contained a single fixed nsSNP between the resequenced cultivated and wild soybean populations. Both differences encode non-conservative amino acid substitutions and may affect protein function (Fig. 3c). In case of Glyma17g14191.1 (homologous to OsMADS56), we observed two conservative amino acid substitutions and one 4 amino acid insertion at a short amino acid span between positions 178 and 183 fixed between the cultivated and wild soybeans. In all the three genes, the fixed amino acid substitutions lie outside of the conserved domains (Fig. 3c), consistent with the previous cloning studies of canonical domestication genes including *tga1*^54^ and *PROG1*.^55^

**Table 2.** Coding sequence diversity and amino acid differences in 10 cultivated and 6 wild resequenced soybean genomes at domestication candidate genes homologous to canonical domestication genes

Domestication gene	Soybean candidate gene	Length (bp)		ROD	*F*_ST_	Genetic diversity in genic region				Genetic diversity in CDS				Amino acid difference^a^ from reference	
						*π* × 10^−3^		*θ_w_* × 10^−3^		*π* × 10^−3^		*θ_w_* × 10^−3^			
		Genic	CDS	100-kb	100-kb	Cul	Wild	Cul	Wild	Cul	Wild	Cul	Wild	Wild (MAF)	Cul (MAF)
*tga1*	*Glyma17g08840.2*	5477	1635	0.982	0.452	0.2	2.42	0.37	0.26	0	0.37	0	0.27	Q491H (0.5)	nd
*qSH1*	*Glyma12g29991.3*	7859	2385	0.994	0.427	0.31	1.88	1.12	0.54	0	1.12	0	1.47	N34D (0.17); P42S (0.17); H240L (0.17); C658T (0.17); N674H (1)	nd
*fw2.2*	*Glyma15g01990.3*	2332	507	0.995	0.667	0	8.75	1.05	0	0	1.05	0	0.86	T648A (0.33)	nd
*PROG1*	*Glyma17g18110.1*	1601	633	0.994	0.493	0.22	3.16	1.05	0.22	0.56	1.05	0.56	1.38	V21_D22insSW (0.17); N76K (0.17)	H16D (0.2)
*DAG1*; *DAG2*	*Glyma16g02550.3*	2343	831	0.987	0.727	0	0.71	0	0	0	0	0	0	nd	nd
*Tunicate1*	*Glyma08g07260.3*	9994	615	0.997	0.470	0.19	2.87	0	0.25	0	0	0	0	nd	nd
*OsMADS56*	*Glyma05g03660.8*	7654	684	0.994	0.542	0.26	5.38	1.27	0.23	0.78	1.27	0.52	1.28	I124V (0.17), T218A (1)	nd
	*Glyma17g14191.1*	8420	663	0.991	0.546	0.002	4.18	1.41	0.004	0	1.41	0	1.32	G177F (1); T180S (1); N182_V183insDAEL (1)	nd
	*Glyma17g08861.1*	5779	1143	0.982	0.452	0.2	2.62	2.9	0.31	0	0.29	0	0.38	nd	nd

Cul, cultivated soybean; Wild, wild soybean; CDS, coding sequence; MAF, minor allele frequency; nd, not detected.

^a^The format for an amino acid difference is X#Y, where X is the amino acid of the Williams 82 reference genome, # is the position of the substitution, and Y is the new amino acid; and X#_Y#insAB, where X and Y are the amino acids of the Williams 82 reference genome, #s are the positions of the insertion, and insAB indicates A and B amino acids were inserted.

We searched for significantly enriched GO terms among genes in CDRs and identified several GO terms reminiscent of domestication-related traits or molecular features of cloned domestication genes (Supplementary Table S17). The candidate genes were enriched in the GO terms related to seed development, morphology, growth, and transcriptional regulation. Interestingly, the terms ‘embryo development ending in seed dormancy’ and ‘response to abscisic acid stimulus’ in the biological process category, which are related to seed germination, were also observed to be enriched terms (*P* < 0.001) in the GO analysis of the lost genes described above. In the molecular function and cellular component categories, several terms such as ‘DNA binding’ and ‘nucleus’, which are related to transcription factors, were enriched (*P* < 0.001). This is somewhat consistent with the fact that the majority of domestication genes obtained by map-based cloning have been transcription factor genes.^53^ Several GO terms related to transport and stress responses, which play important roles in improving yield and stress resistance,^56^ were also identified.

**Figure 4.**
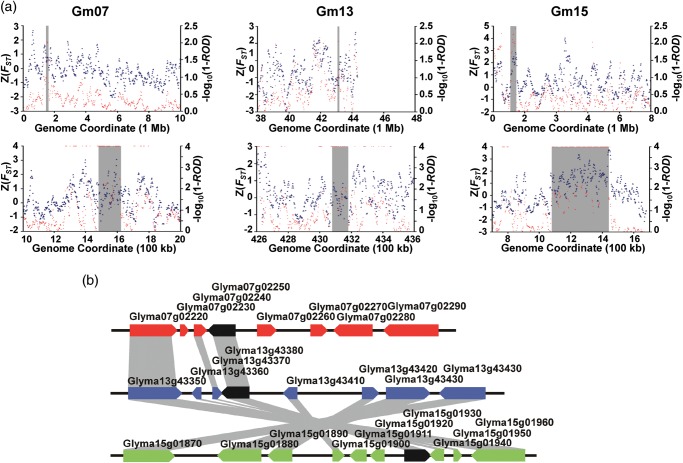
Selection of three ROPGEF12 homeologs. (a) ROD (red), and average fixation index, *F*_ST_ (blue), plotted for 100-kb windows (upper panel) or 10-kb windows (lower panel) across 10-Mb or 1-Mb regions, respectively, of three duplicated chromosomal segments from palaeopolyploidization harbouring a ROPGEF12 homologue. Grey boxes indicate 140-kb (Gm07), 100-kb (Gm13), and 360-kb (Gm15) chromosomal regions having >0.98 ROD values in 100-kb windows and its corresponding region in 10-kb windows. For simplicity, *–*log_10_(1 − ROD) values of ≥4 are shown as corresponding with *–*log_10_(1 − ROD) = 4. (b) Homeologous (duplicated) relationship between genes on the three duplicated chromosomal segments. Predicted genes are indicated by coloured block arrows except black arrows for ROPGEF12 homologues. Grey boxes between genes show homeologs.

Genes regulating plant morphology, to which most of the domestication-related traits belong, typically show pleiotropic effects as exemplified by the ABC model.^57^ These genes may appear in more than two enriched GO terms relevant to domestication-related traits in the biological process category. By searching for candidate domestication genes enriched in multiple GO terms, we found three such genes as *ROPGEF12* (homologous to *Glyma07g02250*, *Glyma13g43380*, and *Glyma15g01930*), *TCP4* (*Glyma12g14200*), and *TCP5* (*Glyma17g08761*) (Supplementary Table S18). *ROPGEF12* is involved in polar growth of pollen tubes in *Arabidopsis*^58^ and is expressed differentially during the pollination process in the maize.^59^ Among the three *ROPGEF12* homologues, *Glyma07g02250* was particularly interesting because its chromosomal location was associated with multiple QTL for plant morphology reported by several independent studies (Supplementary Table S18). The TCP5 homologue, *Glyma17g08761*, was observed in the CDR-containing homologues of *tga1* and *OsMADS56* on Gm17 described above. The soybean genome encodes 57 predicted TCPs. By examining the genes in CDRs, we found two additional TCPs, *Glyma05g03610* and *Glyma17g14160*, whose gene symbols have not been assigned. Our phylogenetic analysis showed that *Glyma12g14200* and *Glyma17g08761* belong to the CIN clade of Class II TCPs, and *Glyma05g03610* and *Glyma17g14160* belong to Class I TCPs^60^ (Fig. 3b and Supplementary Fig. S9). These results suggested that our CDRs do not contain a TCP gene belonging to the CYC/TB1 clade containing the canonical domestication gene *tb1*. Nevertheless, as most of the functionally studied TCPs are involved in cell proliferation and branching, these four soybean genes are strong candidate domestication genes. This notion is further supported by that these genes are associated with multiple QTL for plant morphology (Supplementary Table S18). Interestingly, the three *ROPGEF12* homologues are located on the three duplicated chromosomal segments from palaeopolyploidization^36^ (Fig. 4). Two TCP genes, *Glyma05g03610* and *Glyma17g14160*, whose TCP domains are 100% identical but whose overall identity is 79%, are also located on the duplicated chromosomal segments (Supplementary Fig. S10). These observations likely reflect the important role that co-selection between these ancient duplicated segments has had in soybean domestication.

## 4. Conclusions

In this study, we have provided a large genome variation dataset for the wild and cultivated soybeans. Millions of variations in the representative wild and cultivated soybean strains provided an opportunity to finely resolve the domestication or improvement history of cultivated soybean. The population structure and phylogenetic analyses not only support the hypothesis that cultivated soybeans (*G. max*) are a subclade of their progenitor wild soybeans (*G. soja*), but also disprove the hypothesis of multiple soybean domestication events in East Asia. We identified thousands of candidate genes that may have been artificially selected during the soybean domestication or improvement. The SNPs will be useful as dense markers of genome variation for marker-assisted mapping of important soybean traits as well as for pinpointing agronomically important genes in soybeans. The candidate genes selected during domestication may be agronomically important, and our results generally support that a translational genomics study would be productive for identifying the soybean domestication genes. Taken together with the data from Lam *et al*.^5^ and from Li *et al*.,^61^ which was published after the submission of the present paper with 25 soybean genomes sequenced to a low depth with a mean of 3.38×, the data generated in this study provide a valuable resource for improving soybean as well as elucidating the origin and evolution of soybean.

## Authors' contributions

W.H.C., N.J., N.K., and S.C.J. wrote the article. J.K.M., N.K., and S.C.J. designed the research. W.H.C., J.K., Y.G.L., S.H.L., W.Y., J.H.K., H.K.C., N.K., and S.C.J. carried out analysis of the genome sequences. N.J., W.K.L., I.Y.C., and S.C.J. performed genome sequencing and plant experiments.

## Supplementary Data

Supplementary data are available at www.dnaresearch.oxfordjournals.org.

## Funding

This study was supported principally by a grant from the Next-Generation BioGreen 21 Program (PJ008124), by the Rural Development Administration, and partly by supported by the National Research Foundation of Korea grant funded by the Korea government (no. 20110030770) and by the Korea Research Institute of Bioscience and Biotechnology Research Initiative Program.

## Supplementary Material

Supplementary Data
