# Supplementary material for: Population Structure and Domestication Revealed by High-Depth Resequencing of Korean Cultivated and Wild Soybean Genomes
Source: DNA Res. 2013 Nov 21;21(2):153–67. doi: 10.1093/dnares/dst047 (PMC3989487; doi:10.1093/dnares/dst047)
Supplement: Supplementary Data [file supp_21_2_153__index.html]

Population Structure and Domestication Revealed by High-Depth Resequencing of Korean Cultivated and Wild Soybean Genomes — Population Structure and Domestication Revealed by High-Depth Resequencing of Korean Cultivated and Wild Soybean Genomes — Supplementary Data 

# Population Structure and Domestication Revealed by High-Depth Resequencing of Korean Cultivated and Wild Soybean Genomes

## Supplementary Data

Supplementary Data

**Files in this Data Supplement:**

- Supplementary Data - Supplementary Data
- Supplementary Data - Supplementary Data
- Supplementary Data\_Set1 - zip file
- Supplementary Data\_Set2 - zip file
- Supplementary Data\_Set3 - zip file
