# Supplementary material for: Population Structure and Domestication Revealed by High-Depth Resequencing of Korean Cultivated and Wild Soybean Genomes
Source: DNA Res. 2013 Nov 21;21(2):153–67. doi: 10.1093/dnares/dst047 (PMC3989487; doi:10.1093/dnares/dst047)
Supplement: Supplementary Data [file supp_dst047_dst047supp1.pdf]

Chung *et al.*: Population structure and domestication revealed by high-depth resequencing of Korean cultivated and wild soybean genomes

Supplementary Figures S1 – S10

Supplementary Tables S1 – S7, Tables S11 – S14, Table S16, and Table S18 (Supplementary Table S8, S9, S10, S15, S17 were included in a separate excel file)

Supplementary Data Set 1 - 3

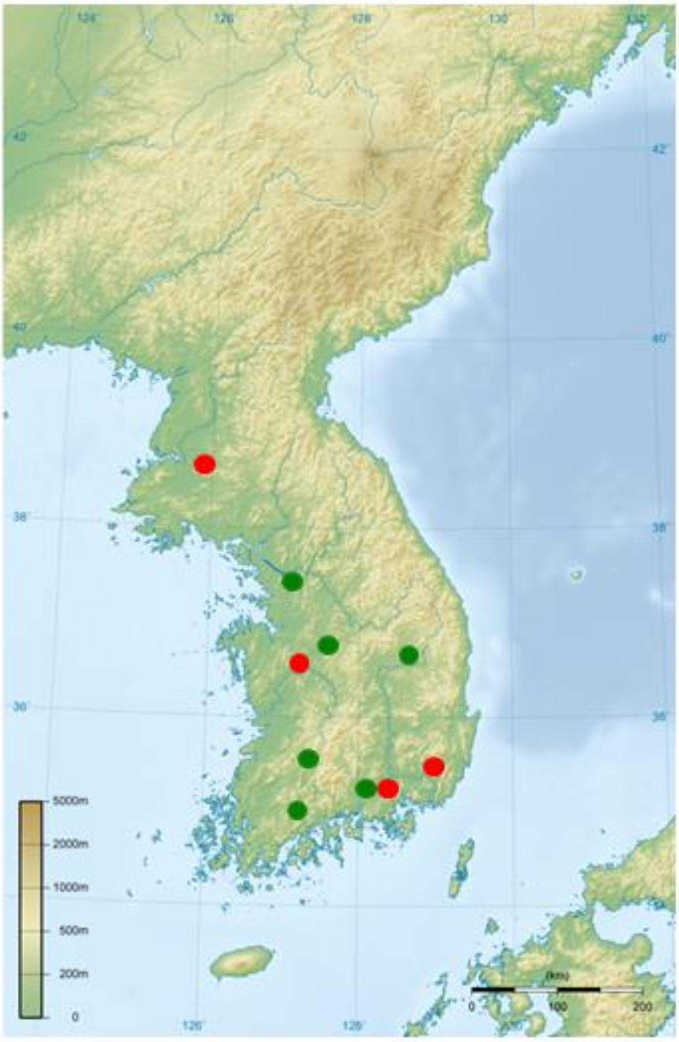

Supplementary Figure S1. Map of the Korean peninsula with soybean landrace accessions (red circles) and wild accessions (green circles) resequenced in this study. This map was downloaded from http://commons.wikimedia.org.

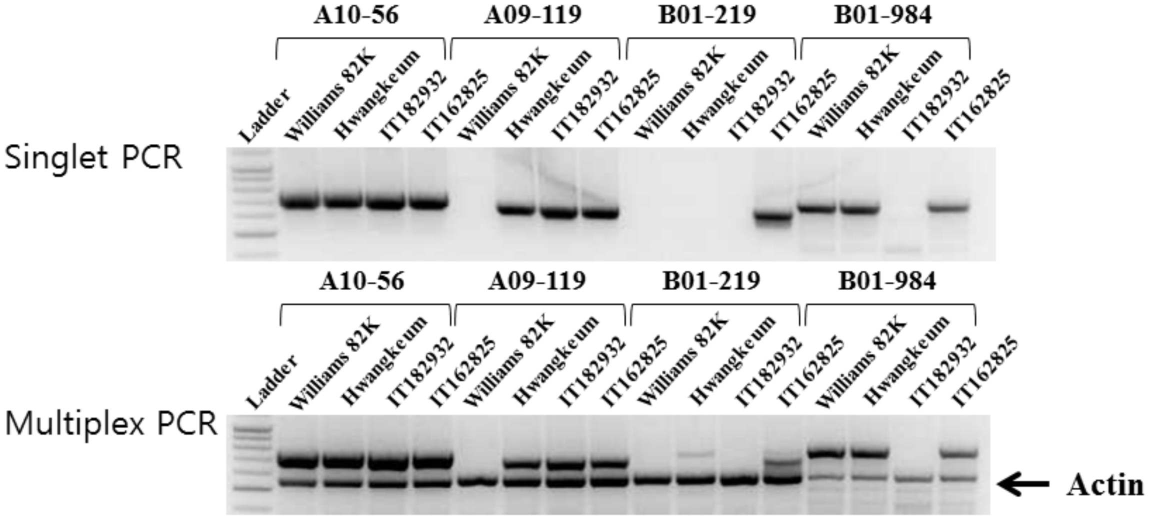

Supplementary Figure S2. Representative agarose gel separation patterns of PCR products for validation of 'novel' genes. 51 novel genes identified in the cultivated soybeans Hwangkeum (8) and Williams 82K (4) and the wild soybean IT162825 (39) were chosen for validation. For each novel gene, primers were designed for PCR-amplifying a specific product ranging from 700 to 800 bp. Genomic DNAs from Williams 82K, Hwangkeum, IT182932, and IT162825 were used as templates. Ladder is 100-bp DNA marker from Bioneer.

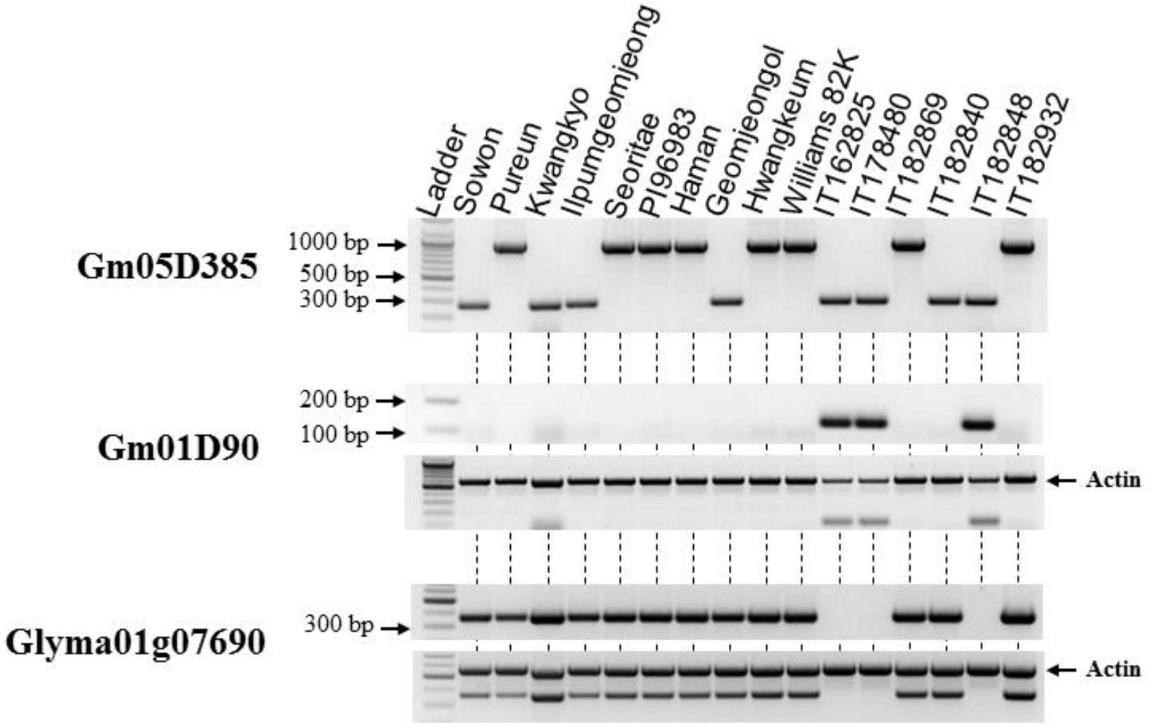

Supplementary Figure S3. Representative agarose gel separation patterns of PCR products for validation of medium-size deletions. 45 medium-size deletions identified in the reseqeunced 16 soybean accessions were chosen for validation. For each deletion, primers were designed for PCR-amplifying a specific product ranging from 200 to 300 bp from both regions flanking its detection site. Genomic DNAs from 16 resequenced soybean accessions were used as templates. Ladder is 100-bp DNA marker from Bioneer. Gm05D385 is a 600-bp deletion site and was PCR-amplified using primers AACTCAACCAGGATCAGATG and GGGAGTCTAGACCCAAAAA. Gm01D90 is a 32,085-bp deletion and was PCR-amplified using primers TGTTGTAAATTTTCCATGGTC and GGCAAATTCCCTAATAGCTT. Glyma01g07690 is a gene located in Gm01D90 and was PCR-amplified using primers GGGCTCACAATAACAAAAG and CCTCTTGCCAATAGTTTCAC. PCR amplification of Glyma01g07690, which is located in deleted region of Gm01D90 and was PCR-amplified using GGGCTCACAATAACAAAAAG and CCTCTTGCCAATAGTTTCAC, was performed to validate deletion of internal part of the 36-kb Gm01D90 deletion. Similarly, Gm01InD379, which is a 54,484 bp deletion, was also confirmed by PCR-amplifying Glyma01g22871 and Glyma01g22880.

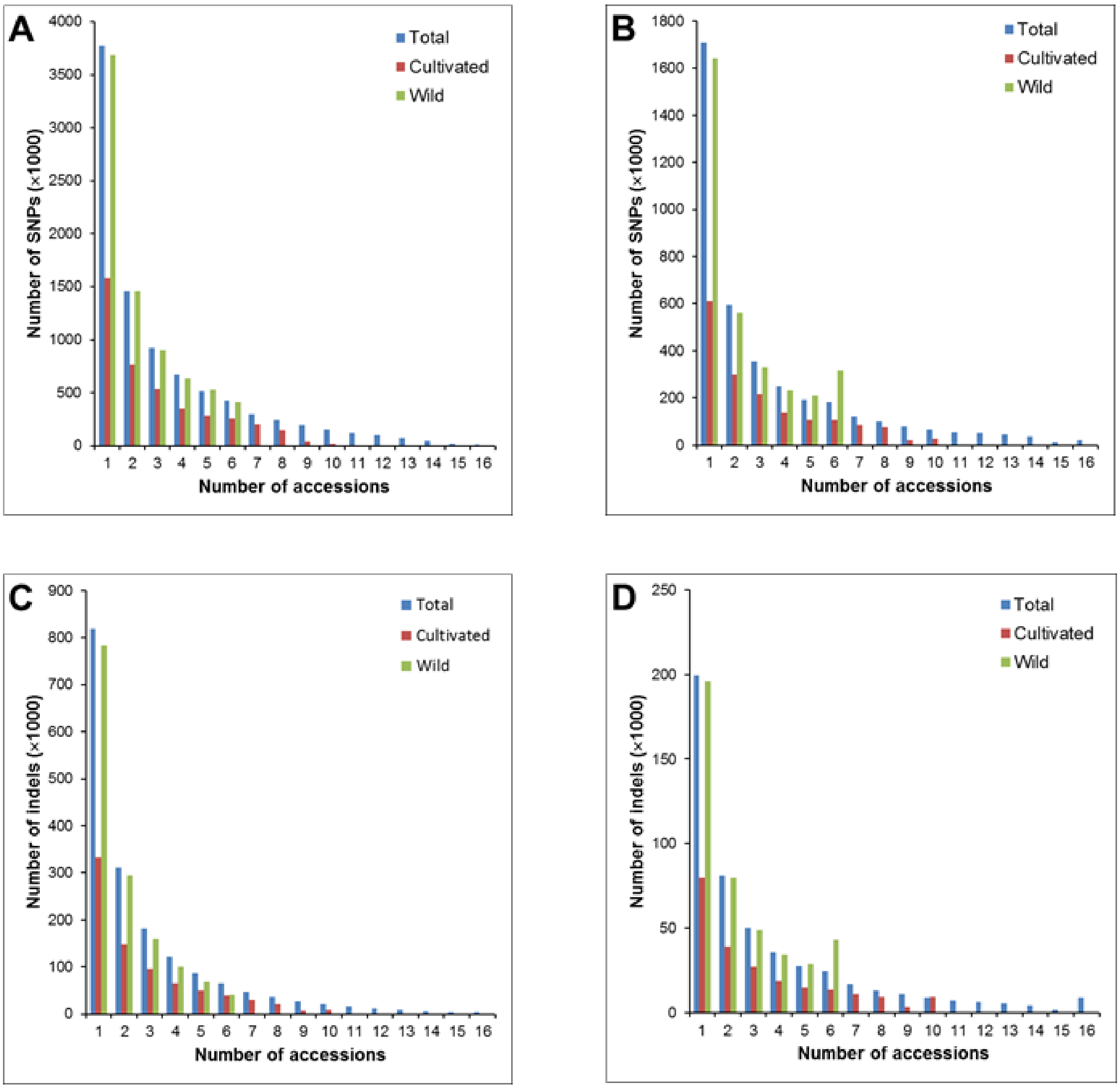

Supplementary Figure S4. Frequency distributions of SNPs and indels within population. The number of accessions sharing the SNPs and indels was calculated. (*A*) Individual genotyping of SNPs. (*B*) Multi-sample genotyping of SNPs. (*C*) Individual genotyping of indels. (*D*) Multi-sample genotyping of indels. The blue bar indicates SNPs or indels in total (whole) population, the red bar indicates those in cultivated accessions, and the green bar indicates those in wild accessions.

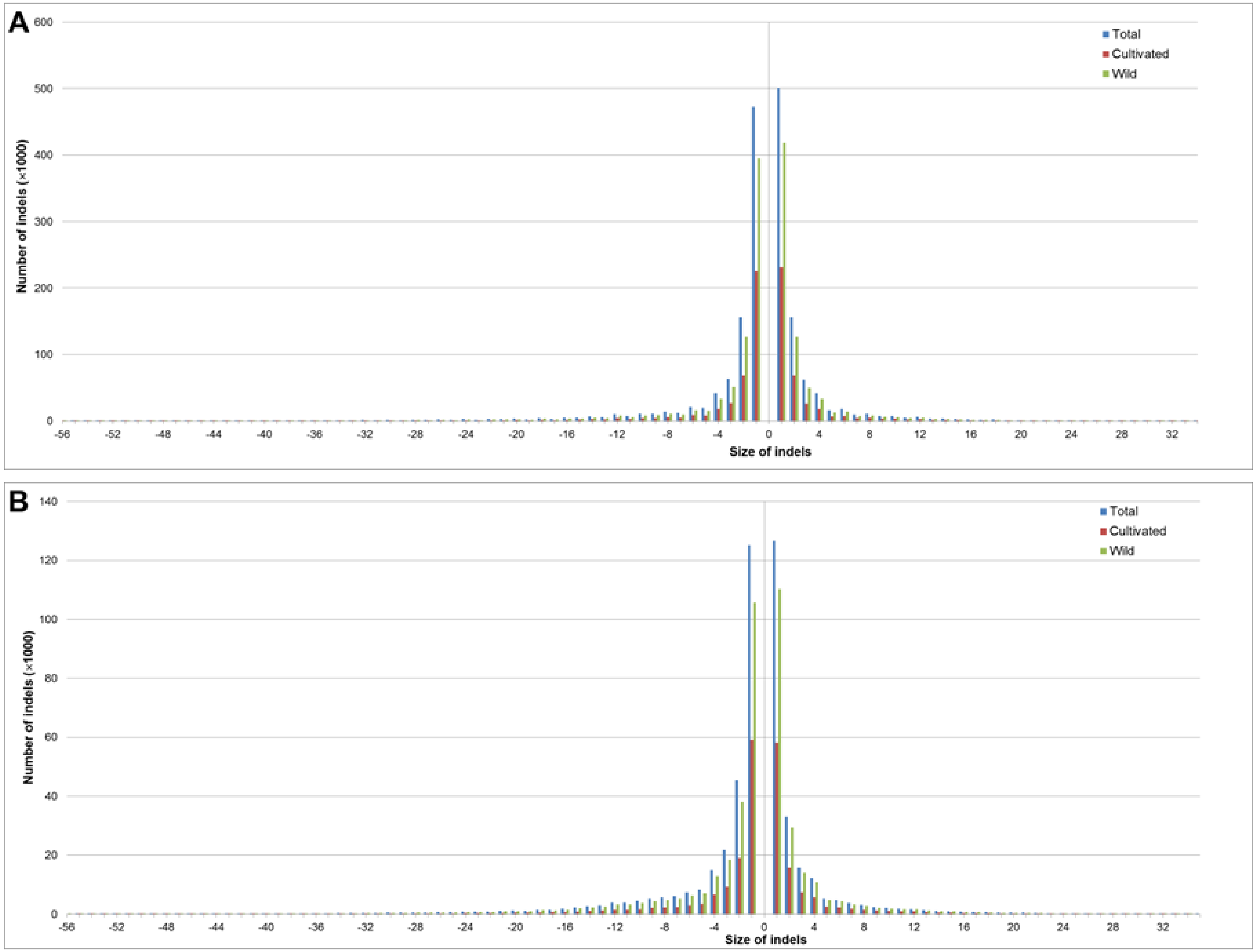

Supplementary Figure S5. Size distribution of redundant indels. The blue bar indicates indels in total (whole) population, the red bar indicates those in cultivated accessions, and the green bar indicates those in wild accessions.

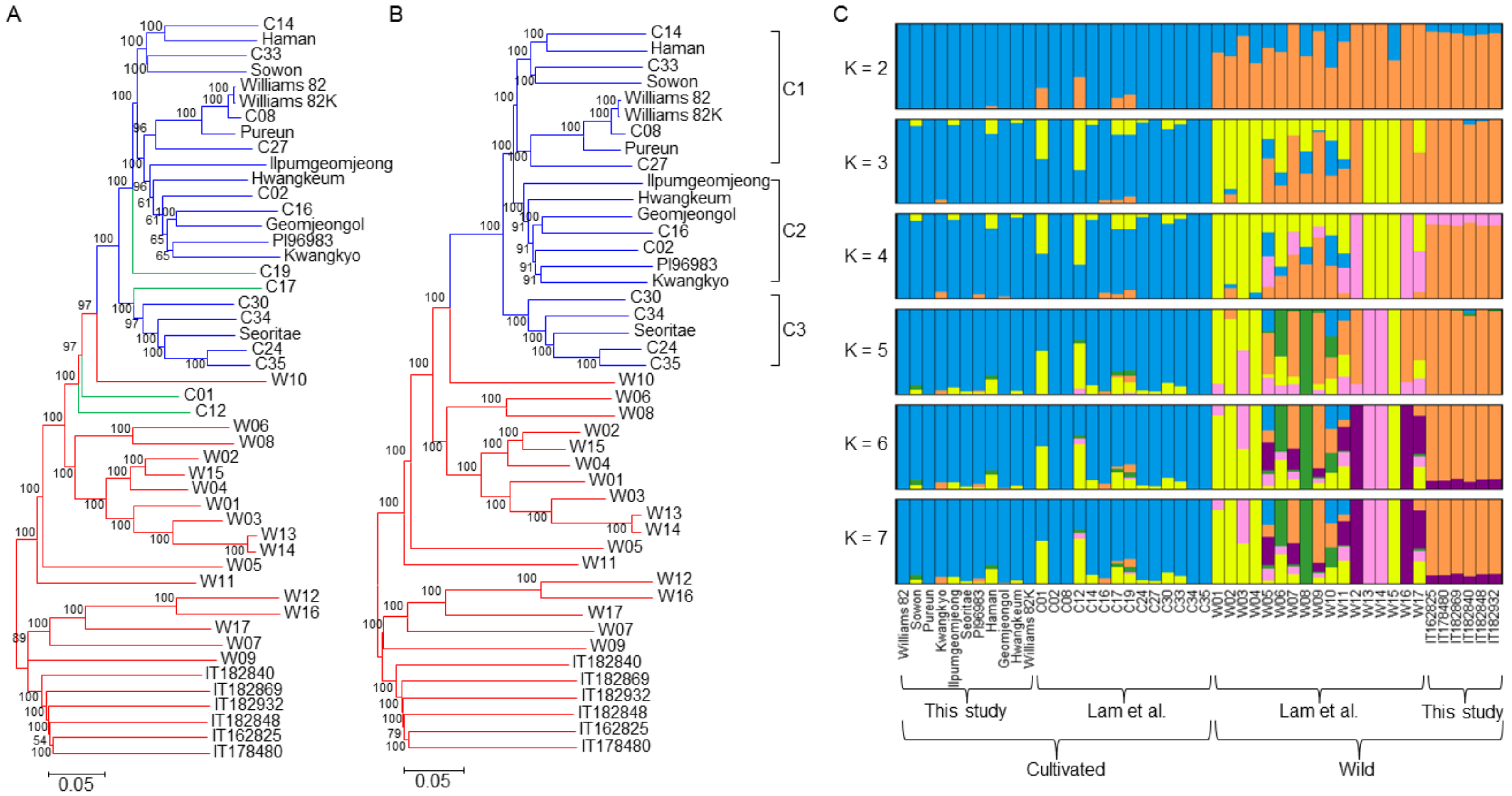

Supplementary Figure S6. Population structure of soybeans with adding 17 wild and 14 cultivated soybean accessions from Lam et al. (2010). We extracted SNPs covered by all 31 accessions without the missing data or heterozygous genotypes. By intersecting these SNPs with the set of 3.3 million high-quality SNPs identified in our 16 accessions, we obtained 208,684 SNPs that could be used to analyze all 23 wild and 24 cultivated soybean accessions. (*A*) Neighbor-joining phylogenetic tree of soybean nuclear genomes based on the high-quality SNPs, with the evolutionary distances measured by *p*-distance. Percent bootstrap value from 1000 bootstrap replications is indicated at each branch. Taxa in the neighbor-joining tree are represented by different colors: wild (red), cultivated (blue), admixture soybeans. (*B*) Neighbor-joining phylogenetic tree of soybean nuclear genomes after excluding four admixture accessions. Cultivated soybeans were tentatively grouped into C1, C2, and C3. (*C*) Bayesian clustering of samples using the STRUCTURE program. Each accession is represented by a vertical bar and each color represents one population. The mean value of ln likelihood when K changed from 2 to 7 was -7841032, -7146017, -6804025, -6546197, -6210216, and -6210216, respectively.

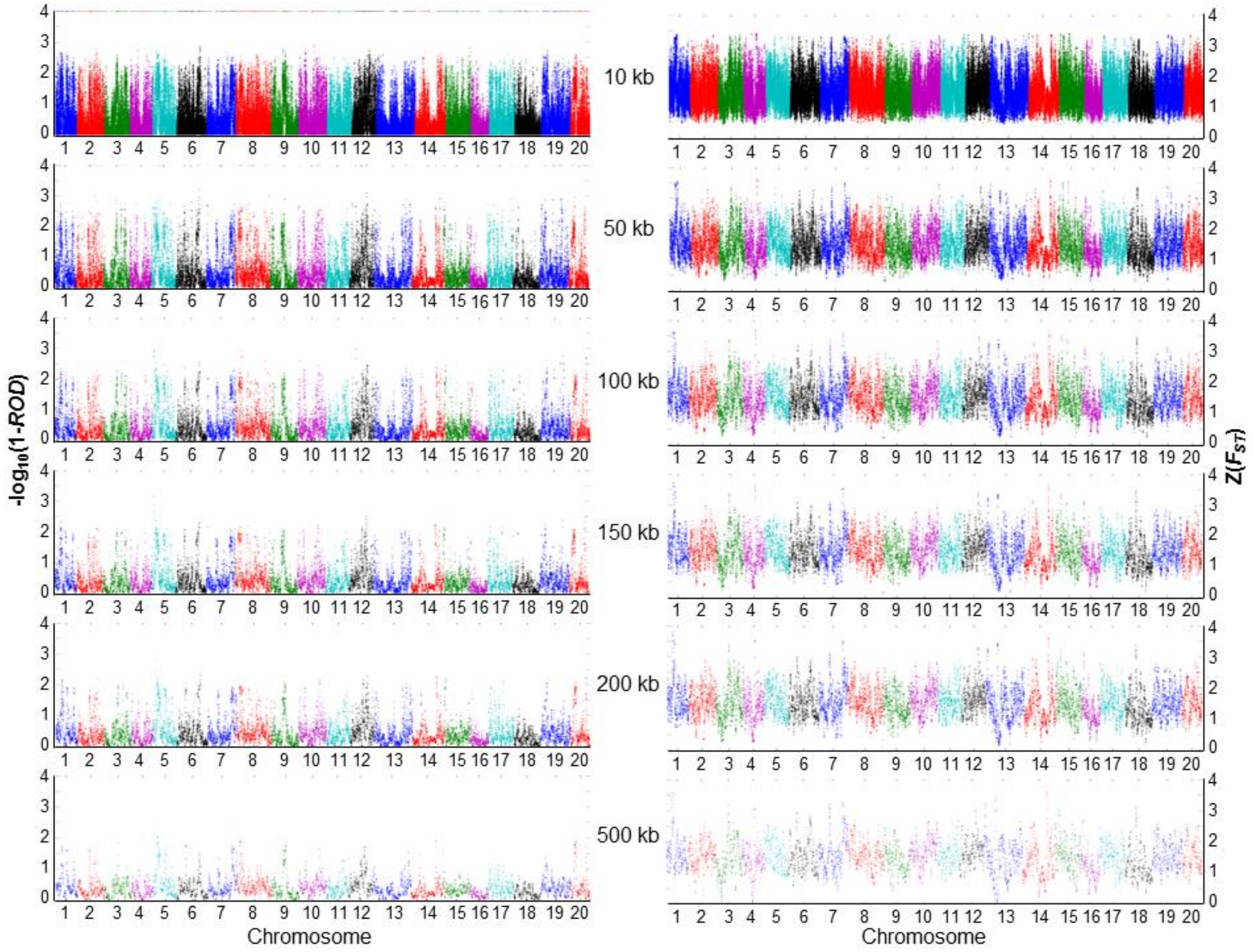

Supplementary Figure S7. Genome-wide *ROD* and F_{ST} distributions. *ROD* distribution for cultivated relative to wild soybeans in 10-kb, 50-kb, 100-kb, 150-kb, 200-kb, and 500-kb windows across the genome (*left panel*). *ROD* = 0.98 corresponds with –log10(1-*ROD*) ≈ 1.70. Distribution of *Z*-transformed F_{ST} values for cultivated relative to wild soybeans in 10-kb, 50-kb, 100-kb, 150-kb, 200-kb, and 500-kb windows across the genome (*right panel*). Overall distribution patterns between different windows are similar to each other, indicating that the regions detected by larger windows are a subset of those detected by smaller windows.

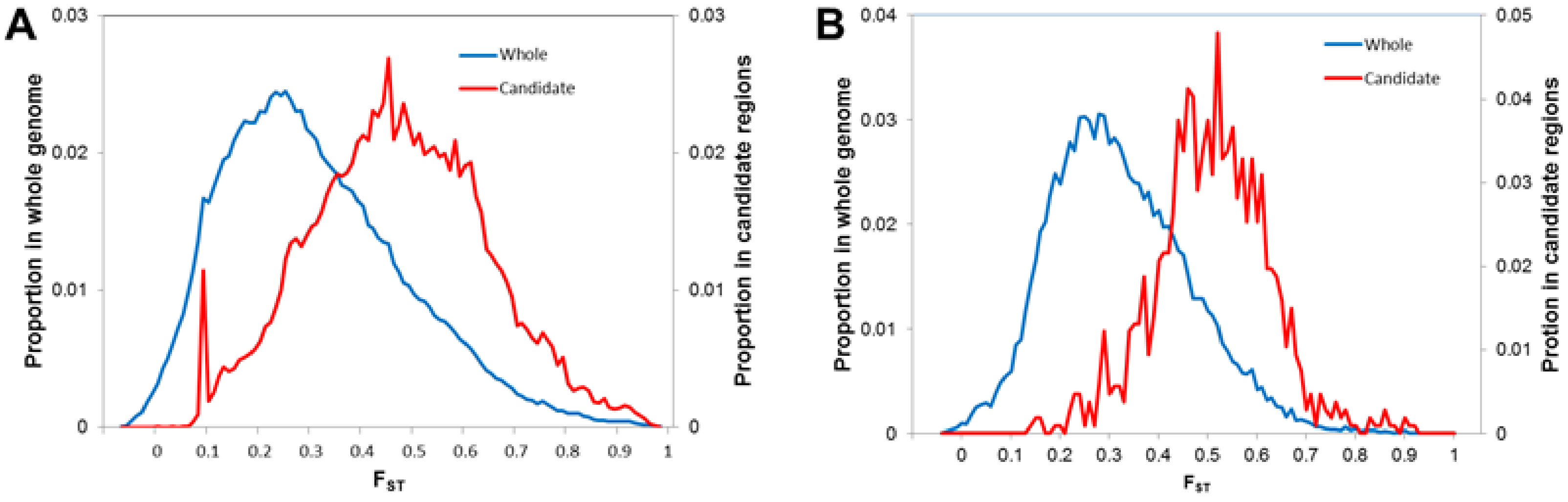

Supplementary Figure S8. F_{ST} distribution of whole genome and candidate regions under selection. The F_{ST} of candidate domestication regions between cultivated and wild soybeans (in red) are compared to the F_{ST} of the whole genome (in blue). Candidate artificial selection regions used here are regions with > 0.98 *ROD* values. (*A*) 10-kb windows. (*B*) 100-kb windows.

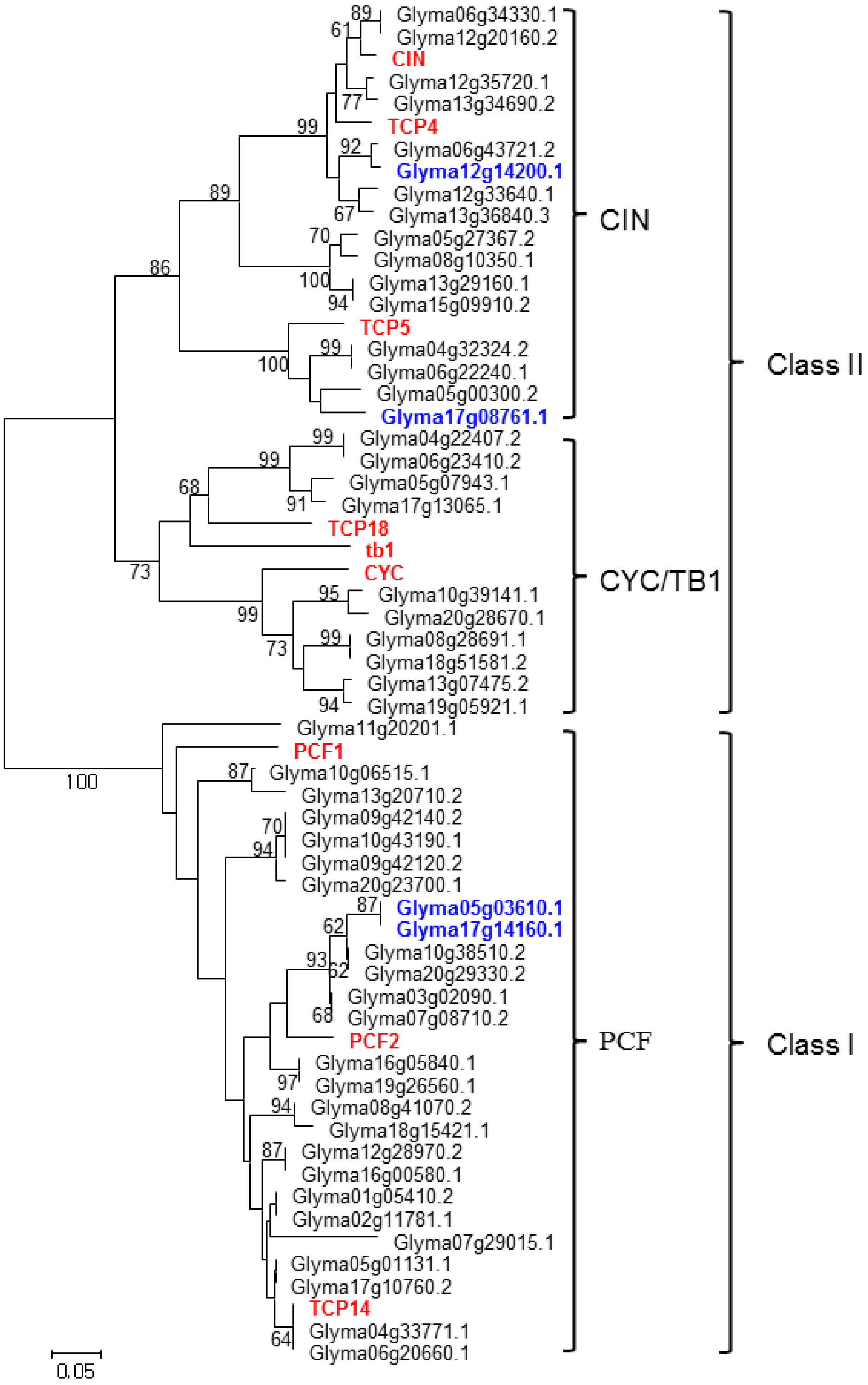

Supplementary Figure S9. Neighbor-joining phylogenetic tree showing relationship of the soybean TCP gene family proteins. Only the TCP domain was used for the analysis. Functionally-characterized representative members of other species are shown in red: TCP4, TCP5, TCP14, and TCP18 from *Arabidopsis thaliana*; CIN and CYC from *Antirrhinum majus*; PCF1 and PCF2 from *Oryza sativa*; tb1 from *Zea mays*. Soybean TCP proteins detected in candidate domestication regions are in blue. More than 60% bootstrap values are shown to indicate the reliability for branching.

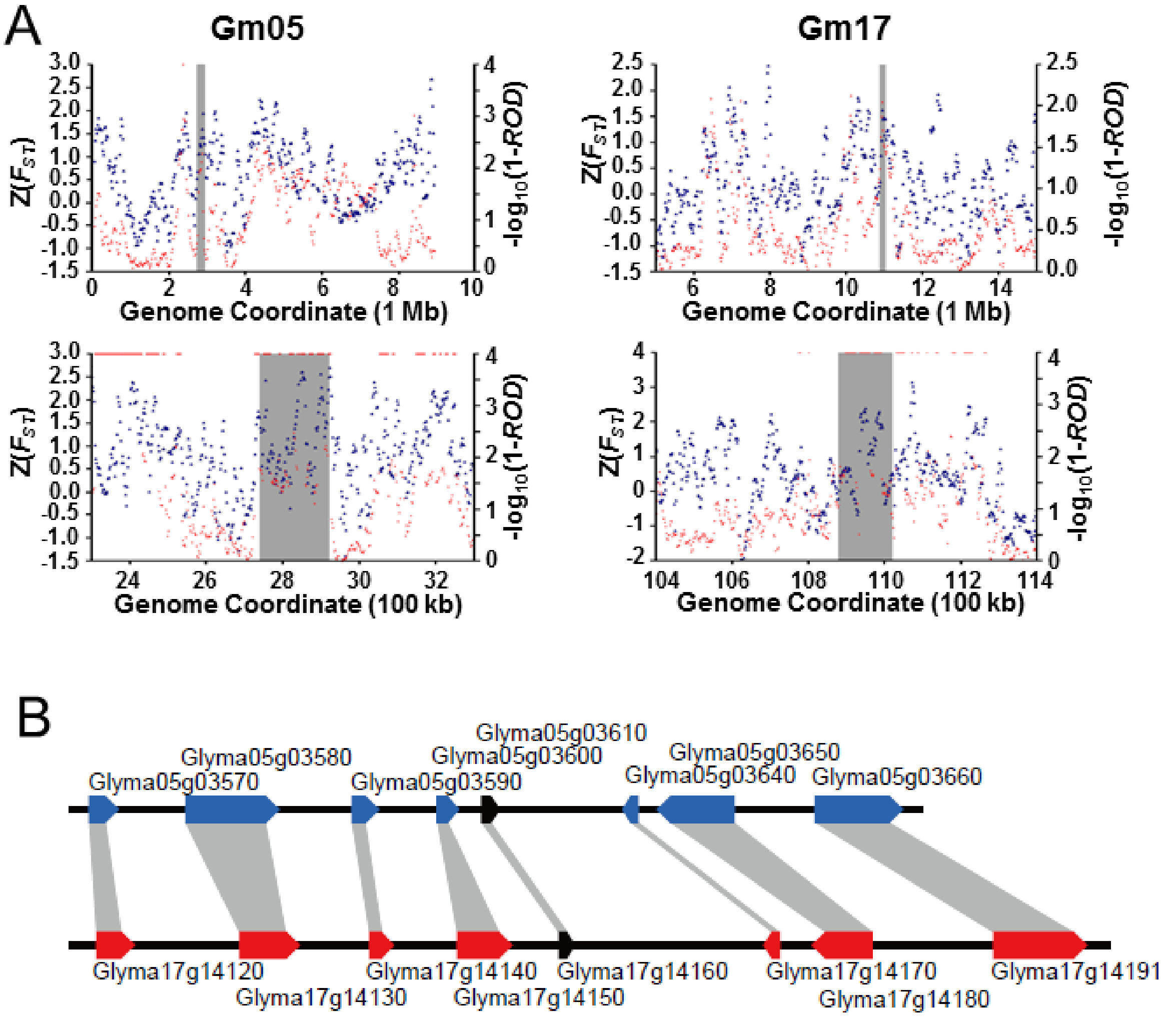

Supplementary Figure S10. Selection of two TCP homologs. (*A*) Reduction of diversity, *ROD* (red), and average fixation index, F_{ST} (blue), plotted for 100-kb windows (upper panel) or 10-kb windows (lower panel) across 10-Mb or 1-Mb regions, respectively, of two duplicated chromosomal segments from palaeopolyploidization harboring a TCP homolog. Gray boxes indicate 180-kb (Gm05) and 140-kb (Gm17) chromosomal regions having > 0.98 *ROD* values in 100-kb windows and its corresponding region in 10-kb windows. For simplicity, $-\log_{10}(1\text{-}ROD)$ values ≥ 4 are shown as corresponding with $-\log_{10}(1\text{-}ROD) = 4$. (*B*) Homeologous (duplicated) relationship between genes on the two duplicated chromosomal segments. Predicted genes are indicated by colored block arrows except black arrows for TCP homologs. Gray boxes between genes show homeologs.

Supplementary Table S1. Summary of soybean accessions and resequencing

Accession code	Accession	Description[1]	Food use[2]	Raw reads depth[3]	Mapped reads (%)	Non-dup. depth[3,4]	Genome coverage (%)		
							≥ 1 reads	≥ 5 reads	≥ 10 reads
A01	Sowon	Advanced bred line	Sprout	30.2	95.3	25.2	98.2	94.8	80.8
A02	Pureun	Advanced bred line	Sprout	29.4	96.0	25.3	99.0	96.8	83.2
A03	Kwangkyo	Advanced bred line	Sauce-tofu	22.7	95.4	17.9	98.0	95.4	87.2
A04	Ilpumgeomjeong	Advanced bred line	Cooking-with-rice	17.9	94.7	14.4	98.2	94.7	78.7
A05	Seoritae	Landrace; Cheongwon, Korea	Cooking-with-rice	19.7	95.2	16.2	98.4	95.5	83.8
A06	PI96983	Landrace; Shariin, Korea	Sauce-tofu	19.0	95.4	16.1	98.2	95.4	84.0
A07	Haman	Landrace; Haman, Korea	Sauce-tofu	22.0	96.5	19.2	98.2	96.2	90.2
A08	Geomjeongol	Landrace; Milyang, Korea	Green	17.0	96.0	14.7	98.3	95.1	80.2
A09	Hwangkeum	Advanced bred line	Sauce-tofu	20.8	96.7	18.0	98.5	96.5	89.3
A10	Williams 82K	Advanced bred line	Sauce-tofu	19.8	97.7	17.5	99.7	98.8	90.7
B01	IT162825	Wild; Yecheon, Korea	NA	19.9	94.4	17.0	97.5	94.7	87.2
B02	IT178480	Wild; Boeun, Korea	NA	22.4	95.7	19.8	97.5	94.7	88.0
B03	IT182869	Wild; Jinju, Korea	NA	19.7	95.6	17.3	97.5	94.7	87.3
B04	IT182840	Wild; Imsil, Korea	NA	21.1	95.3	18.5	97.4	94.7	88.7
B05	IT182848	Wild; Gokseong, Korea	NA	19.1	95.8	16.8	97.6	95.0	87.5
Gsoja	IT182932	Wild; Yongin, Korea	NA	41.8	86.3	30.1	96.1	85.4	74.5

[1]The wild soybeans were collected from different provinces in South Korea.

[2]Green, sprout, sauce-tofu, and cooking-with-rice soybeans, which are four groups traditionally classified in terms of food use in Korea, are indicated to each of cultivated soybeans. NA, not applicable

[3]Genome size used to compute read depth: 950,068,807 bp

[4]Non-dup.: non-duplicated reads

Supplementary Table S2. Breeding history of pure bred accessions

Accession	Parents	Ancestors of maternal lines	Ancestors of paternal lines	Breeding method
Sowon	Bangsa x Eunha	CB27 (pedigree unknown)	N48-1248, Perry, Patoka, L37-1355, CNS, S-100, S5-7075, Lee, D69-7816, Essex	Pedigree breeding
Pureun	Cheongsaek Namul (Korean landrace) x L78-379 (Williams(6) x PI 96983)	NA	Wayne, L57-0034 (Clark x Adams), Handarin, Manchu, Lincoln, Richland, Illini, Dunfield, CNS	Pedigree breeding
Kwangkyo	Jangdan Baekmok (Korean landrace) x Yukwoo 3 (Japanese landrace)	NA	NA	Pedigree breeding
Ilpumgeomjeong	SLSB83-3 (Taiwan landrace) x YS558	NA	Kwangkyo, Clark63, SS7006, Baekmokjang Yeop, Hwangkeum, SLSB45	Pedigree breeding
Hwangkeum	(F_2 Kwangkyo x Clark63) x Baekmokjang Yeop (Japanese landrace)	Jangdan Baekmok, Yukwoo 3, Handarin, Manchu, Lincoln, Richland, Clark, CNS, Mukden, Blackhawk	NA	Pedigree breeding
Williams 82K	Williams(7) x Kingwa (Chinese landrace)	Wayne, L57-0034 (Clark x Adams), Handarin, Manchu, Lincoln, Richland, Illini, Dunfield, CNS	NA	Backcross breeding

NA, not applicable as it is a landrace

Supplementary Table S3. Summary of the sequenced reads

Accession code	Insert size (bp)	Total bases (Gb)	Raw reads[a] (M)	Mapped reads[b] (M)	% of mapped (=b/a*100)	Reads in chromosome[c] (M)	% of in chromosome (=c/a*100)	Non-duplicated reads[d] (M)	% of non-duplicated (=d/a*100)
A01	237	28.6	283.6	270.1	95.3	249.9	88.1	236.6	83.4
A02	241	28.0	276.9	265.5	96.0	246.1	88.9	237.8	85.9
A03	239	21.6	213.8	204.1	95.4	184.5	86.3	168.6	78.9
A04	245	17.0	168.3	159.3	94.7	144.2	85.7	135.6	80.6
A05	246	18.7	185.5	176.6	95.2	161.6	87.1	152.8	82.4
A06	239	18.0	178.4	170.3	95.4	157.5	88.3	151.5	84.9
A07	240	20.9	206.5	199.3	96.5	183.0	88.6	180.7	87.5
A08	276	16.2	160.2	153.7	96.0	141.5	88.3	138.0	86.1
A09	250	19.8	195.6	189.1	96.7	172.9	88.4	169.4	86.6
A10	256	18.8	186.3	182.1	97.7	167.2	89.8	164.8	88.5
B01	336	18.9	187.5	177.0	94.4	163.1	87.0	160.0	85.3
B02	239	21.3	211.0	202.0	95.7	188.8	89.5	186.3	88.3
B03	266	18.7	185.0	176.8	95.6	165.5	89.5	163.0	88.1
B04	269	20.1	198.6	189.3	95.3	177.4	89.3	174.4	87.8
B05	275	18.2	180.1	172.5	95.8	162.1	90.0	158.5	88.0
IT182932[1]	271	39.8	523.1	451.3	86.3	398.1	76.1	376.2	71.9

[1]Illumina 76 bp paired-ends; the others are Illumina 101 bp paired-ends
a: million number of sequenced reads
b: million number of mapped reads onto the Williams 82
c: million number of reads mapped onto the chromosomes (Gm01~Gm20)
d: million number of non-duplicated reads; used for SNP/Indel genotyping

Supplementary Table S4. Heterochromatic regions. Definition: Continuous and gene-poor pericentrometric regions having < 0.286 cM/Mb recombination rates and/or containing <25 genes per 1 Mbp flanking putative centeromeres. A genetic map in an F_{15} population derived from a cross between Hwangkeum and IT182932 (Lee et al. 2013[a]) was used.

Chromosome	Pericentromeric region (Mb)
Gm01	8.4..41.9
Gm02	15.6..38.9
Gm03	8.5..27.8
Gm04	11.4..38.2
Gm05	9.0..25.8
Gm06	20.5..40.3
Gm07	19.1..34.1
Gm08	23.3..34.1
Gm09	10.1..29.1
Gm10	12.6..34.4
Gm11	18.5..32.1
Gm12	15.6..31.2
Gm13	14.9..19.6
Gm14	11.0..27.8
Gm15	18.1..42.1
Gm16	8.0..25.5
Gm17	15.8..31.3
Gm18	12.8..47.2
Gm19	8.8..28.7
Gm20	3.9..30.0

[a]Lee WK, Kim N, Kim J, Moon J-K, Jeong N, Choi I-Y, Kim SC, Chung W-H, Kim HS, Lee S-H, et al. 2013. Dynamic genetic features of chromosomes revealed by comparison of soybean genetic and sequence-based physical maps. *Theor Appl Genet* **126:**1103–1119.

Supplementary Table S5. Estimation of genome sizes of soybean accessions using distribution peak values of 17-mer frequency in the raw sequencing reads

Accession	read count	k-mer	high peak	low peak	genome size
Sowon	171,321,390	17	13	4	1,094,144,403
Pureun	167,311,416	17	13	4	1,067,751,119
Kwangkyo	213,843,518	17	16	5	1,108,585,312
Ilpumgeomjeong	168,312,212	17	12	4	1,159,902,017
Seoritae	185,546,598	17	14	5	1,099,609,713
PI96983	178,443,244	17	14	4	1,057,997,510
Haman	206,537,918	17	15	5	1,127,002,326
Geomjeongol	160,170,956	17	12	4	1,089,715,237
Hwangkeum	195,559,384	17	14	5	1,157,153,024
Williams 82K	186,311,324	17	14	5	1,103,397,549
IT162825	187,485,388	17	13	5	1,182,710,994
IT178480	211,030,946	17	15	5	1,161,394,541
IT182869	184,960,670	17	14	5	1,089,194,939
IT182840	198,619,808	17	15	5	1,091,528,440
IT182848	180,111,166	17	14	5	1,058,385,508
IT182932	274,715,532	17	15	4	1,053,951,280

Supplementary Table S6. Summary of the unmapped reads assembly and annotation

Accession	Unmapped reads size (Mb)	Unmapped (%)	Contigs (≥2kb)				Annotated genes (≥100 a.a)		
			Count	Size (kb)	Maximum	L50	Count	Maximum	Mean
Sowon	978.5	5.7	516	1,610.3	11,082	3,139	102	1,265	281
Pureun	863.1	5.1	356	1,090.0	11,006	3,016	60	1,494	264
Kwangkyo	1,252.5	5.8	672	2,177.4	12,143	3,231	179	1,077	271
Ilpumgeomjeong	1,164.6	6.9	435	1,300.4	9,179	2,926	87	992	287
Seoritae	1,126.4	6.0	518	1,628.1	11,741	3,090	112	1,450	285
PI96983	1,042.1	5.8	543	1,690.8	9,001	3,082	121	1,251	284
Haman	892.5	4.3	553	1,768.8	13,260	3,167	191	1,396	376
Geomjeongol	810.1	5.0	532	1,657.4	9,753	3,059	137	956	280
Hwangkeum	756.3	3.8	514	1,595.1	15,667	3,045	178	1,562	398
Williams 82K	524.6	2.8	263	854.4	8,612	3,182	40	1,569	305
IT162825	1,378.9	7.3	1,052	3,341.1	14,300	3,199	352	1,352	315
IT178480	1,131.9	5.3	946	2,942.3	14,735	3,107	344	1,519	388
IT182869	1,032.1	5.5	985	3,070.9	13,127	3,103	288	1,478	312
IT182840	1,154.1	5.8	973	3,114.5	14,531	3,169	310	1,346	336
IT182848	943.7	5.2	894	2,790.5	11,475	3,085	241	1,392	325
IT182932	6,269.8	3.8	283	830.6	9,073	2,832	202	1,595	498
Total	21,321.2	4.7	10,035	31,462.5	15,667	3,112	2,944	1,595	338

Supplementary Table S7. Summary of the gene annotation of the contigs from the unmapped reads

Name	All genes	Non-redundant (%)	NCBI NR BLAST search
Count	2,944	1,363 (46.3)	343
Size (a.a)	996,017	518,104 (52.0)	131768
Min (a.a)	100	100	100
Max (a.a)	1,595	1,595	1569
Mean (a.a)	338	380	384

Supplemental Data Set1. Annotation information of 'gain' genes identified from unmapped contigs. Data available as two fasta files in online supplementary information.

Supplemental Data Set2. Sequences flanking the identified medium-size deletion sites. Data available as a fasta file in online supplementary information.

Supplemental Data Set3. List of soybean non-singleton (common) SNPs. Data available as a plain text file in online supplementary information.

Supplementary Table S8. List of coding sequences, contig sequences, BLAST search results, and presence/absence genotypes of gain genes obtained from de novo assembly of reads of each of 16 soybean resequencing data, which were unmapped to the soybean reference genome sequence. Table available as an excel file in online.

Supplementary Table S9. List of medium-size deletions and lost genes. Table available as an excel file in online.

Supplementary Table S10. Significantly enriched gene ontology (GO) terms among lost genes (GO biological process terms related to domestication-related traits are in red). Table available as an excel file in online.

Supplementary Table S11. Genes that show differential frequency between cultivated and wild soybeans among lost genes in significantly enriched gene ontology (GO) terms related to domestication-related traits

Biological process GO term	Gene symbol	Deletion ID	Glyma ID	Function description	Number of accessions containing this gene loss event		
					Total	Cultivar	Wild
Embryo development ending in seed dormancy	TTN9	Gm18D877	Glyma18g45446	Titan9	6	1	5
	EMB2744	Gm19D28	Glyma19g02725	Pentatricopeptide repeat (PPR) superfamily protein	5	0	5
	OEP80	Gm13D437	Glyma13g41560	Outer envelope protein of 80 kDa	3	0	3
Pollen germination	PAP15	Gm11D342	Glyma11g36510	Purple acid phosphatase 15	6	6	0
	GSL10	Gm20D573	Glyma20g38860	Glucan synthase-like 10	6	1	5
Response to red or far red light	FRS5	Gm13D162	Glyma13g10260	FAR1-related sequence 5	6	1	5
Abscisic acid mediated signaling pathway	PUB9	Gm18D563	Glyma18g31330	Plant U-box 9	7	7	0
Megagametogenesis	RHF2A	Gm12D71	Glyma12g08186	RING-H2 group F2A	4 (5)[a]	0	4 (5)
Flower development	HAC12	Gm15D1	Glyma15g00241	Histone acetyltransferase of the CBP family 12	3 (4)	0	3 (4)
	LUG	Gm14D168	Glyma14g16040	LisH dimerisation motif; WD40/YVTN repeat-like-containing domain	4	0	4

[a] Number in parentheses indicates changed number of deletion events by one accession miscalling in each of two deletions, which were validated by PCR (**Supplementary Table S8**).

Supplementary Table S12. Distribution of SNPs and indels in different soybean accessions

Multi-sample genotyping	SNP					Indel				
type	total	homozygous	heterozygous	mixed	CDS	total	homozygous	heterozygous	mixed	CDS
Sowon	547811	520302	27509	0	24622	73655	68495	5160	0	1387
Pureun	225202	207088	18114	0	12167	36969	33677	3292	0	903
Kwangkyo	701410	668240	33170	0	33314	94704	88160	6544	0	1745
Ilpumgeomjeong	602145	573483	28662	0	30229	84463	78705	5758	0	1687
Seoritae	741470	551654	189816	0	36671	102635	77134	25501	0	1916
PI96983	606547	578878	27669	0	30235	85884	80446	5438	0	1630
Haman	561976	536202	25774	0	26099	77044	72288	4756	0	1471
Geomjeongol	626663	598793	27870	0	31575	88680	83417	5263	0	1682
Hwangkeum	534063	508102	25961	0	27048	75863	71041	4822	0	1514
Williams 82K	98443	20128	78315	0	5755	22143	8403	13740	0	682
IT162825	1284381	1236785	47596	0	58546	175579	164649	10930	0	2924
IT178480	1281748	1235319	46429	0	58438	175350	164760	10590	0	2795
IT182869	1284017	1237117	46900	0	58801	175633	165131	10502	0	2938
IT182840	1275779	1230165	45614	0	58426	174242	163886	10356	0	2838
IT182848	1243529	1197616	45913	0	57584	172396	162200	10196	0	2785
IT182932	1263836	1209064	54772	0	59119	167210	154404	12806	0	2818
Cultivar	1687232	1388531	119511	179190	78172	225609	176166	20813	28630	3972
Wild type	3290830	3151577	102570	36683	147121	430564	396067	20875	13622	6824
Total	3871469	3480027	145135	246307	173293	499865	426296	25424	48145	8222

Individual genotyping	SNP					Indel				
type	total	homozygous	heterozygous	mixed	CDS	total	homozygous	heterozygous	mixed	CDS
Sowon	1245409	1189560	55849	0	38488	216243	206708	9535	0	1986
Pureun	605925	561686	44239	0	21726	116665	110759	5906	0	1366

Kwangkyo	1689398	1603260	86138	0	56034	300020	284617	15403	0	2782
Ilpumgeomjeong	1367346	1296885	70461	0	49148	244807	233726	11081	0	2367
Seoritae	1649999	1280794	369205	0	58451	295985	238707	57278	0	2780
PI96983	1471792	1390614	81178	0	51510	265581	252656	12925	0	2575
Haman	1330112	1257977	72135	0	44651	231053	221368	9685	0	2486
Geomjeongol	1474614	1401118	73496	0	51874	269522	258094	11428	0	2465
Hwangkeum	1279135	1201594	77541	0	46284	227923	217515	10408	0	2588
Williams 82K	128540	54485	74055	0	5190	30988	21562	9426	0	742
IT162825	2964174	2849687	114487	0	97163	508390	487387	21003	0	4457
IT178480	2938653	2822513	116140	0	99860	501178	481374	19804	0	4768
IT182869	2969165	2853288	115877	0	99198	512537	492254	20283	0	4788
IT182840	2972259	2858527	113732	0	98879	515502	495299	20203	0	4795
IT182848	2889082	2774515	114567	0	98033	510158	489613	20545	0	4582
IT182932	2237941	2132199	105742	0	97528	291654	276905	14749	0	4638
Cultivar	4182059	3533804	389419	258836	139107	799470	687577	63774	48119	7269
Wild type	7626486	7166363	366725	93398	252245	1447750	1359836	66646	21268	13003
Total	9028250	8049055	560741	418454	296648	1769260	1586699	100329	82232	15764

Supplementary Table S13. Pairwise distances among seventeen soybean accessions

	1	2	3	4	5	6	7	8	9	10	11	12	13	14	15	16
1. Williams 82																
2. Sowon	0.159															
3. Pureun	0.046	0.152														
4. Kwangkyo	0.213	0.191	0.203													
5. Ilpumgeomjeong	0.165	0.174	0.158	0.163												
6. Seoritae	0.175	0.185	0.167	0.197	0.184											
7. PI96983	0.179	0.165	0.166	0.154	0.167	0.177										
8. Haman	0.162	0.165	0.161	0.212	0.186	0.192	0.164									
9. Geomjeongol	0.181	0.176	0.173	0.142	0.160	0.168	0.158	0.191								
10. Hwangkeum	0.149	0.172	0.159	0.150	0.163	0.178	0.162	0.180	0.137							
11. Williams 82K	0.006	0.155	0.041	0.209	0.160	0.170	0.174	0.157	0.176	0.146						
12. IT182932	0.475	0.486	0.475	0.462	0.478	0.480	0.468	0.489	0.472	0.466	0.469					
13. IT162825	0.498	0.503	0.492	0.487	0.494	0.491	0.481	0.500	0.491	0.493	0.491	0.476				
14. IT178480	0.499	0.513	0.495	0.501	0.502	0.498	0.494	0.519	0.498	0.496	0.492	0.456	0.454			
15. IT182869	0.496	0.489	0.492	0.478	0.487	0.483	0.473	0.500	0.480	0.480	0.490	0.465	0.480	0.471		
16. IT182840	0.494	0.490	0.487	0.471	0.487	0.486	0.481	0.497	0.487	0.487	0.488	0.467	0.490	0.480	0.488	
17. IT182848	0.473	0.477	0.469	0.466	0.467	0.477	0.475	0.499	0.474	0.475	0.466	0.451	0.455	0.449	0.471	0.495

Supplementary Table S14. Percentage of >0.98 *ROD* regions in upper 25% and 10% tails of F_{ST} distribution based on *ROD* and F_{ST} values calculated in six sliding windows

	F_{ST}	
Window	25% tail	10% tail
10kb	62.0	31.2
50kb	76.3	41.8
100kb	81.5	50.0
150kb	84.1	56.6
200kb	86.4	60.3
500kb	94.1	73.5

Supplementary Table S15. List of soybean domestication candidate genes detected in candidate domestication regions (CDRs). Table available as an excel file in online.

Supplementary Table S16. The summary of public putative domestication-trait QTL associated with soybean candidate domestication genes homologous to canonical domestication genes

Canonical Domestication gene					Soybean candidate domestication gene					
							Amino acid identity (%) to canonical protein		Public QTL near the soybean candidate domestication gene	
Name	Gene symbol	Crop	Molecular and Phenotypic Function	Causative change (references[a])	Soybean candidate domestication gene	Position on soybean chromosome	Overall	Conserved domain (name)		References[a] for QTL
tga1		Maize	Transcriptional regulator (SBP); seed casing	Amino acid change (S1)	Glyma17g08840.2	Gm17:6,526,544..6,532,020	23	72 (SBP)	Leaflet length, plant weight; seed weight, yield	S2, S3, S4, S5, S6
qSH1	BLH8, PNF	Rice	Transcriptional regulator (homeodomain); abscission layer formation, shattering	Regulatory change (S7)	Glyma12g29991.3	Gm12:33,462,050..33,469,908	26	84 (homeodomain)	Seed yield	S8
fw2.2		Tomato	Cell signaling; fruit weight	Regulatory change (S9)	Glyma15g01990.3	Gm15:1,306,383..1,308,714	33		Leaflet shape, pod dehiscence	S3, S10
PROG1	ZFP7	Rice	Transcriptional regulator; Prostrate growth	Amino acid change (S11)	Glyma17g18110.1	Gm17:15,317,414..15,319,014	20	63 (C2H2 zinc finger)	Lodging	S12

DAG1 and DAG2	DAG1	Maize	Transcriptional regulator; germination	Knockout mutant (S13, S14, S15)	Glyma16g02550.3	Gm16:2,119,565..2,121,907	35/37	88/90 (Dof domain)	Not available	
Tunicate1	AGL22, SVP	Maize	Transcriptional regulator (MADS box); pod corn	Regulatory change (S16)	Glyma08g07260.3	Gm08:5,215,213..5,225,206	43	70	Pod number, leaflet shape	S3, S17
OsMADS56	AGL3, SEP4	Rice	Transcriptional regulator; flowering	Knockout mutant (S15, S18)	Glyma05g03660.8	Gm05:2,820,385..2,828,038	46	92	Pod number	S17
					Glyma17g14191.1	Gm17:10,961,514..10,969,933	46	90	Seed weight, seed yield	S19, S20, S21
					Glyma17g08861.1	Gm17:6,559,991..6,565,769	36	82	Leaflet length, plant weight; seed weight, yield	S2, S3, S4, S5, S6

[a]References

S1. Wang H, Nussbaum-Wagler T, Li B, Zhao Q, Vigouroux Y, Faller M, Bomblies K, Lukens L, Doebley JF. 2005. The origin of the naked grains of maize. *Nature* **436:** 714-719.

S2. Zhang W, Wang Y, Luo G, Zhang J, He C, Wu X, Gai J, Chen S. 2004. QTL mapping of ten agronomic traits on the soybean (*Glycine max* L. Merr) genetic map and their association with EST markers. *Theor Appl Genet* **108:**1131-1139.

S3. Kim H, Kang S, Suh D. 2005. Analysis of quantitative trait loci associated with leaflet types in two recombinant inbred lines of soybean. *Plant Breeding* **124:** 582-589.

S4. Gai J, Wang Y, Wu X, Chen S. 2007. A comparative study on segregation analysis and QTL mapping of quantitative traits in plants-with a case in soybean. *Front of Ag*

in China 1: 1-7.

S5. Liu B, Fujita T, Yan Z-H, Sakamoto S, Xu D, Abe J. 2007. QTL mapping of domestication-related traits in soybean (*Glycine max*). *Ann Bot* (Lond) **100:** 1027–1038.

S6. Lian Q, Xiaohui C, Mantong M, Xiaolong Y, Hong L. 2010. QTL analysis of root traits as related to phosphorus efficiency in soybean. *Ann Bot* (Lond) **106:** 223-234.

S7. Konishi S, Izawa T., Lin SY, Ebana K, Fukuta Y, Sasaki T, Yano M. 2006. An SNP caused loss of seed shattering during rice domestication. *Science* 312, 1392-1396.

S8. Specht JE, Chase K, Macrander M, Graef GL, Chung J, Markwell JP, Germann M, Orf JH, Lark KG. 2001. Soybean response to water: A QTL analysis of drought tolerance. *Crop Sci* **41:** 493-509.

S9. Frary A, Nesbitt TC, Grandillo S, Knaap E, Cong B, Liu J, Meller J, Elber R, Alpert KB, Tanksley SD. 2000. *fw2.2*: a quantitative trait locus key to the evolution of tomato fruit size. *Science* **289:** 85-88.

S10. Bailey MA, Mian MAR, Carter TE Jr., Ashley DA, Boerma HR. 1997. Pod dehiscence of soybean: trait identification of quantitative trait loci. *J Hered* **88:**152-154.

S11. Tan L, Li X, Liu F, Sun X, Li C, Zhu Z, Fu Y, Cai H, Wang X, Xie D, Sun C. 2008. Control of a key transition from prostrate to erect growth in rice domestication. *Nat Genet* **40:** 1360-1364.

S12. Reinprecht Y, Poysa V, Yu K, Rajcan I, Ablett G, Pauls K. 2006. Seed and agronomic QTL in low linolenic acid, lipoxygenase-free soybean (*Glycine max* (L.) Merrill) germplasm. *Genome* **49:** 1510-1527.

S13. Gualberti G, Papi M, Bellucci L, Ricci I, Bouchez D, Camilleri C, Costantino P, Vittorioso P. 2002. Mutations in the Dof zinc finger genes *DAG2* and *DAG1* influence with opposite effects the germination of *Arabidopsis* seeds. *Plant Cell* **14:** 1253–1263.

S14. Papi M, Sabatini S, Bouchez D, Camilleri C, Costantino P, Vittorioso P. 2000. Identification and disruption of an *Arabidopsis* zinc finger gene controlling seed germination. *Genes Dev* **14:** 28–33.

S15. Hufford MB, Xu X, van Heerwaarden J, Pyhäjärvi T, Chia J-M, Cartwright RA, Elshire RJ, Glaubitz JC, Guill KE, Kaeppler SM, Lai J, Morrell PL, Shannon LM, Song C, Springer NM, Swanson-Wagner RA, Tiffin P, Wang J, Zhang G, Doebley J, McMullen MD, Ware D, Buckler ES, Yang S, Ross-Ibarra J. 2012. Comparative population genomics of maize domestication and improvement. *Nat Genet* **44:** 808-811.

S16. Han JJ, Jackson D, Martienssen R. 2012. Pod corn is caused by rearrangement at the *Tunicate1* locus. *Plant Cell* **4:** 2733-2744.

S17. Zhang D, Cheng H, Wang H, Hengyou Z, Liu C, Yu D. 2007. Identification of genomic regions determining flower and pod numbers development in soybean (*Glycine max* L). *J Genet Genom* **37:** 545-556.

S18. Ryu C-H, Lee S, Cho L-H, Kim SL, Lee Y-S, Choi SC, Jeong HJ, Yi J, Park SJ, Han C-D, An G. 2009. *OsMADS50* and *OsMADS56* function antagonistically in

regulating long day (LD)-dependent flowering in rice. *Plant Cell Environ* 32: 1412–1427.

S19. Orf JH, Chase K, Jarvik T, Mansur LM, Cregan PB, Adler FR, Lark KG. 1999. Genetics of soybean agronomic traits: I. Comparison of three related recombinant inbred populations. *Crop Sci* **39:** 1642-1651.

S20. Reyna N, Sneller CH. 2001. Evaluation of marker-assisted introgression of yield QTL alleles into adapted soybean. *Crop Sci* **41:** 1317-1321.

S21. Panthee DR, Pantalone VR, West DR, Saxton AM, Sams CE. 2005. Quantitative trait loci for seed protein and oil concentration, and seed size in soybean. *Crop Sci* **45:** 2015-2022.

Supplementary Table S17. Significantly enriched gene ontology (GO) terms among domestication candidate genes (GO biological process terms related to domestication-related traits are in red). Table available as an excel file in online.

Supplementary Table S18. Soybean Domestication candidate genes that belong to more than two enriched gene ontology (GO) terms relevant to domestication-related traits in the biological process category

Gene symbol	Enriched GO terms	Glyma ID	Position on soybean chromosome	Function description	Public QTL near the soybean candidate domestication gene	References[a] for QTL
ROPGEF12	embryo development ending in seed dormancy; pollen tube growth	Glyma07g02250	Gm07:1,553,456.. 1,556,901	RHO guanyl-nucleotide exchange factor 12	Seed weight; yield/height; first flower; seed number; yield; seed set; embryo abortion; plant height; pod muturity date; leaflet width;	S22, S23, S24, S25
		Glyma13g43380	Gm13:43,101,685.. 43,104,909	RHO guanyl-nucleotide exchange factor 12	Yield	S25
		Glyma15g01930	Gm15:1,267,892.. 1,270,998	RHO guanyl-nucleotide exchange factor 12	Leaflet legth	S22
TCP4	embryo development ending in seed dormancy; pollen tube growth; leaf morphogenesis	Glyma12g14200	Gm12:12,985,348.. 12,986,868	TEOSINTE BRANCHED 1, cycloidea and PCF (TCP) transcription factor 4	Leaflet area, leaflet width, seed weight	S26, S27
TCP5	multicellular organismal development; leaf morphogenesis	Glyma17g08761	Gm17:6,450,810.. 6,453,048	TCP transcription factor 5	Leaflet length, seed weight	S24, S28, S29
Not assigned		Glyma05g03610	Gm05:2,790,429.. 2,792,018	TCP transcription factor	Pod number; R7 Beginning Maturity; R3 Beginning pod; Seed weight; seed weight per plant	S30, S31, S32

Not assigned	Glyma17g14160	Gm17:10,922,221..10,923,629	TCP transcription factor	Yield/Height; seed weight; yield	S22, S33, S34, S35

The GO term 'regulation of transcription, DNA-dependent' was excluded because most of cloned canonical domestication are transcription factor genes; two additional candidate domestication TCP genes whose gene symbols are not assigned are also listed.

[a]References

S22. Orf JH, Chase K, Jarvik T, Mansur LM, Cregan PB, Adler FR, Lark KG. 1999. Genetics of soybean agronomic traits: I. Comparison of three related recombinant inbred populations. *Crop Sci* **39:** 1642-1651.

S23. Tischner T, Allphin L, Chase K, Orf JH, Lark KG. 2003. Genetics of seed abortion and reproductive traits in soybean. *Crop Sci* **43:** 464-473.

S24. Zhang W, Wang Y, Luo G, Zhang J, He C, Wu X, Gai J, Chen S. 2004. QTL mapping of ten agronomic traits on the soybean (*Glycine max* L. Merr) genetic map and their association with EST markers. *Theor Appl Genet* **108:** 1131-1139.

S25. Specht JE, Chase K, Macrander M, Graef GL, Chung J, Markwell JP, Germann M, Orf JH, Lark KG. 2001. Soybean response to water: A QTL analysis of drought tolerance. *Crop Sci* **41:** 493-509.

S26. Mansur LM, Orf JH, Chase K, Jarvik T, Cregan PB, Lark KG. 1996. Genetic mapping of agronomic traits using recombinant inbred lines of soybean. *Crop Sci* **36:** 1327-1336.

S27. Li D, Pfeiffer T, Cornelius P. 2008. Soybean QTL for yield and yield components associated with *Glycine soja* alleles. *Crop Sci* 2008, 48:571-581.

S28. Chapman A, Pantalone VR, Usten A, Allen FL, Landau-Ellis D, Trigiano RN, Gresshoff PM. 2003. Quantitative trait loci for agronomic and seed quality traits in an F-2 and F-4:6 soybean population. *Euphytica* **129:** 387-393.

S29. Kim H, Kang S, Suh D. 2005. Analysis of quantitative trait loci associated with leaflet types in two recombinant inbred lines of soybean. *Plant Breeding* **124:** 582-589.

S30. Zhang D, Cheng H, Wang H, Hengyou Z, Liu C, Yu D. 2010. Identification of genomic regions determining flower and pod numbers development in soybean (*Glycine max* L). *J Genet Genom* **37:** 545-556.

S31. Tasma IM, Lorenzen LL, Green DE, Shoemaker RC. 2001. Mapping genetic loci for flowering time, maturity, and photoperiod insensitivity in soybean. *Mol Breed* **8:** 25-35.

S32. Chen Q, Zhang Z, Liu C, Xin D, Qiu H, Shan D, Shan C, Hu G. 2007. QTL analysis of major agronomic traits in soybean. *Ag. Sci. in China* **6:** 399-405.

S33. Hoeck JA, Fehr WR, Shoemaker RC, Welke GA, Johnson SL, Cianzio SR. 2003. Molecular marker analysis of seed size in soybean. *Crop Sci* **43:** 68-74.

S34. Reyna N, Sneller CH. 2001. Evaluation of marker-assisted introgression of yield QTL alleles into adapted soybean. *Crop Sci* **41:** 1317-1321.

S35. Panthee DR, Pantalone VR, West DR, Saxton AM, Sams CE. 2005. Quantitative trait loci for seed protein and oil concentration, and seed size in soybean. *Crop Sci* **45:** 2015-2022.
